# Persulfate-activated charcoal mixture: an efficient oxidant for the synthesis of sulfonated benzo[*d*][1,3]oxazines from *N*-(2-vinylphenyl)amides and thiols in aqueous solution†

**DOI:** 10.1039/d1ra02377b

**Published:** 2021-04-27

**Authors:** Palani Natarajan, Priya, Deachen Chuskit

**Affiliations:** Department of Chemistry & Centre for Advanced Studies in Chemistry, Panjab University Chandigarh – 160014 India pnataraj@pu.ac.in

## Abstract

A series of 2,4-aryl-4-((arylsulfonyl)methyl)-4*H*-benzo[*d*][1,3]oxazines in good to excellent yields have directly been obtained from *N*-(2-vinylphenyl)amides and thiols by employing a mixture of K_2_S_2_O_8_-activated charcoal in aqueous acetonitrile solution at 50 °C. A plausible mechanism for the reaction is reported. It reveals that the reaction follows a radical pathway and the persulfate has been the oxygen source for formation of the sulfone group in the products. It is worth mentioning that this protocol utilizes an easily accessible K_2_S_2_O_8_-activated charcoal mixture and thiols, respectively, as an oxidant and sulfonylating precursors for the first time.

## Abstract 2

A simple method for the synthesis of 2,4-aryl-4-((arylsulfonyl)methyl)-4*H*-benzo[*d*][1,3]oxazines using an easily accessible K_2_S_2_O_8_-activated charcoal mixture and thiols, respectively, as an oxidant and sulfonylating precursors has been described.

## Graphical abstract

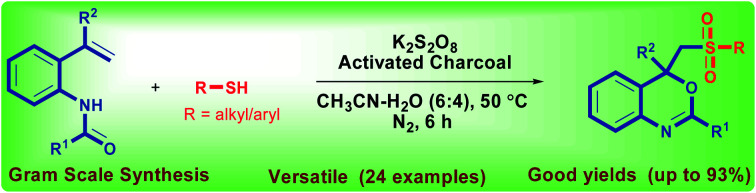

## Introduction

**Scheme 1:**
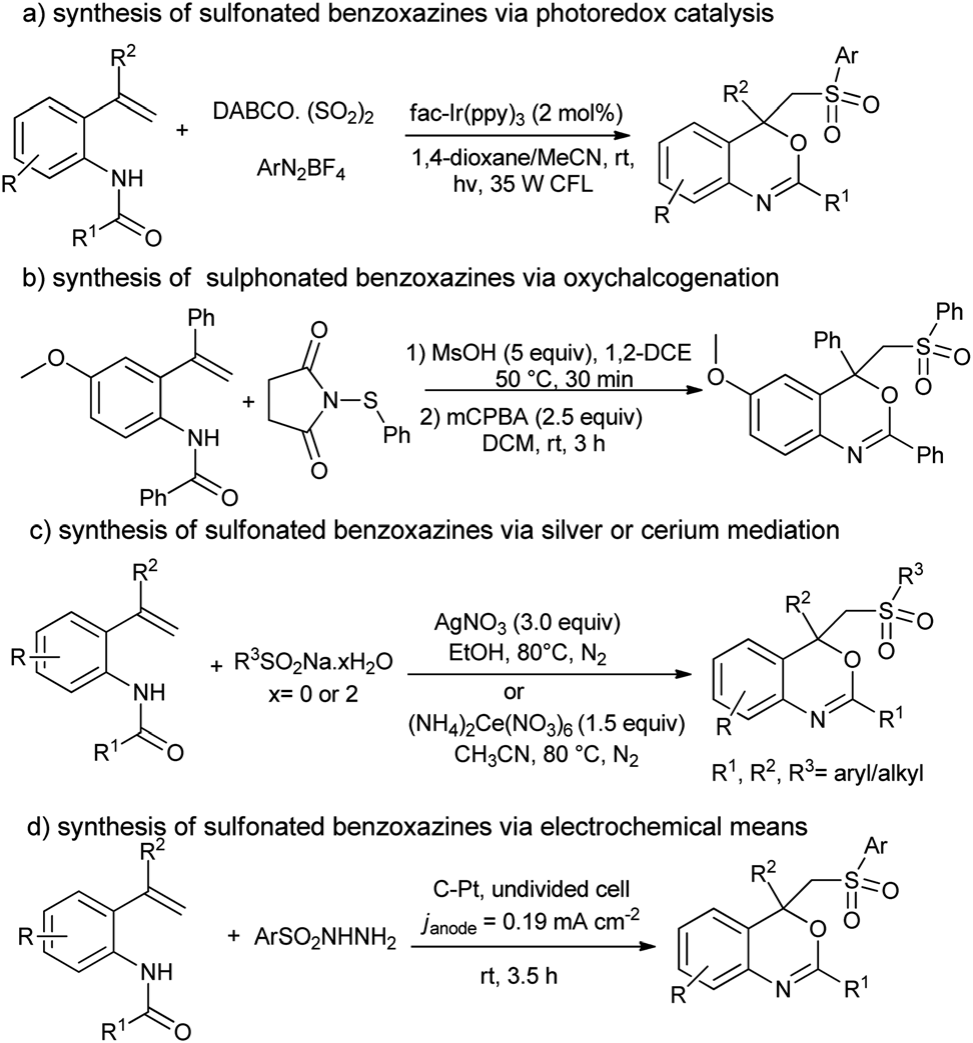
Various protocols for the synthesis of sulfonated benzo[*d*][1,3]oxazines yet reported in literature.

Benzoxazines and their derivatives are an important class of heterocycles frequently found in many natural products^1^ and biologically active compounds^2^ (*e.g.* 6-chloro-*N*-ethyl-4-methyl-4-phenyl-4*H*-benzo[*d*][1,3]oxazin-2-amine and 2-chloro-1-(2,2,4,4-tetramethyl-2*H*-benzo[*d*][1,3]oxazin-1(4*H*)-yl)ethanone). Likewise, as the key structural motif changes the physical and chemical properties of the parent molecule, the sulfone (–SO_2_–) functionality has widely been installed in a variety of functional materials^3^ (pristine polysulfone networks) and drugs^4^ (dapsone, diazoxide and sulfisoxazole). Therefore, development of a practical method for the synthesis of sulfonated benzo[*d*][1,3]oxazines has drawn significant attention from chemists, pharmacists and biologists. In 2018, Wu and co-workers^5^ reported visible-light photocatalysis for the preparation of sulfonated benzo[*d*][1,3]oxazines from *N*-(2-vinylphenyl)amides, DABCO·(SO_2_)_2_ and diazonium salts (Scheme 1a).^5^ Similarly, the acid-mediated oxythiolation of *o*-vinylanilides with *N*-(arylthio)-succinimides and *m*-CPBA was reported by the Anbarasan group in 2018 (Scheme 1b).^6^ In 2019, Li and co-workers^7^ disclosed a metal-catalyzed approach for the synthesis of sulfonated benzo[*d*][1,3]oxazines from *N*-(2-vinylphenyl)amides and alkyl(aryl)sulfinates (Scheme 1c).^7^ Last year, Huang *et al.*^8^ described an electrochemical method for the synthesis of sulfonated benzo[*d*][1,3]oxazines from *N*-(2-vinylphenyl)amides and sulfonyl hydrazines, *cf.*Scheme 1d.^8^

**Scheme 2:**
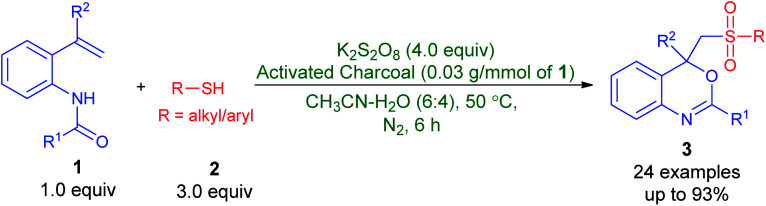
The synthesis of 2,4-aryl-4-((arylsulfonyl)methyl)-4*H*-benzo[*d*][1,3]oxazines from *N*-(2-vinylphenyl)amides and thiols employing mixture of K_2_S_2_O_8_-activated charcoal in aqueous acetonitrile solutional 50 °C reported in this work.

Persulfate (S_2_O_8_^2−^, also known as peroxydisulfate or peroxodisulfate) as readily available and efficient oxidizing reagent has extensively been utilized for organic synthesis^9^ and environmental remediation^10^ in recent decades. Persulfate is a white crystalline solid, cheap, chemically stable at ambient conditions, easy-to handle and convenient to transport. Moreover, upon activation^11^ by heat, metal ions, carbon materials, base, or ultra-violet radiation, persulfate affords a powerful one-electron oxidant such as sulfate anion-radical (SO_4_˙^−^, *E*° = 2.5–3.1 V) that has longer life span than HO˙ precursors, hydrogen peroxide and ozone. In spite of this potentiality, to the best of our knowledge, the use of persulfate for the synthesis of sulfonated benzo[*d*][1,3]oxazines has never been reported. In view of this and in continuation of our ongoing attention to explore the applications of persulfate,^12^ herein we disclose our findings on the preparation of 2,4-aryl-4-((arylsulfonyl)methyl)-4*H*-benzo[*d*][1,3]oxazines from *N*-(2-vinylphenyl)amides and thiols by employing mixture of K_2_S_2_O_8_-activated charcoal in aqueous acetonitrile solution at 50 °C (Scheme 2). Furthermore, a plausible mechanism for the reaction is reported, *vide infra*. Especially, this is a simple and highly efficient method for the construction of C–S, C–O and S–O bonds in one step and utilizes, at the first time, easily accessible K_2_S_2_O_8_-activated charcoal mixture and thiols, respectively, as an oxidant and sulfonylating precursors.

## Results and discussion

We chose *N*-(2-(1-phenylvinyl)phenyl)benzamide (1a, 1.0 mmol) and benzenethiol (2a, 2.0 mmol) as model substrates to optimize conditions for this reaction, and the obtained results are summarized in Table 1. By using 2.0 mmol of K_2_S_2_O_8_ as an oxidant and CH_3_CN/H_2_O (6 : 4, v/v mL) as a solvent, the mixture of 1a and 2a was heated at 80 °C under nitrogen gas atmosphere for 36 h. Fortunately, the expected sulfonated oxindole, *i.e.*, 2,4-diphenyl-4-((phenylsulfonyl)methyl)-4*H*-benzo[*d*][1,3]oxazine (3aa) was produced in 26% yield (Table 1, entry 1). The product 3aa was isolated and characterized by NMR and mass analysis (ESI). Encouraged by this result, 2.0 equiv., of different oxidants include Na_2_S_2_O_8_, (NH_4_)_2_S_2_O_8_, hydrogen peroxide and di-*tert*-butyl peroxide (DTBP) (Table 1, entries 2–5) were tested, and K_2_S_2_O_8_ was found to be the best choice (Table 1, entry 1). Thus, K_2_S_2_O_8_ was taken as an oxidant for further all optimizations and reactions. Replacing reaction medium CH_3_CN/H_2_O (6 : 4, v/v mL) with other common solvents such as methanol, acetonitrile, DMSO, water and EtOAc–H_2_O (5 : 5, v/v mL) could also afforded the desired product 3aa, but in poor yield (Table 1, entries 6–10). To improve the product yield, we then investigated the stoichiometry of K_2_S_2_O_8_ to substrate *N*-(2-(1-phenylvinyl)phenyl)benzamide (1a). A slightly improved yield (43%) of 3aa was noticed by increasing the amount of K_2_S_2_O_8_ to 4.0 equiv. (Table 1, entries 11–13); however further raising the amount of K_2_S_2_O_8_ did not improve the reaction efficiency drastically (Table 1, entries 14–15). Under similar conditions, the reaction gave 54% of product (3aa) with higher quantity of benzenethiol (3.0 equiv., Table 1, entry 16). Additional optimizations revealed that the reaction atmosphere was crucial for outcome of the reaction. The reaction proceeded efficiently under nitrogen gas atmosphere while the involvement of air or molecular oxygen brings down yield of 3aa, *cf.* entries 18 and 19 in Table 1. To further improve the product yield, we decided to activate the persulfate by a mild protocol. As the productivity of sulfate radicals from persulfate can be influenced by the activation types.

**Table 1:** Selected results of screening the optimal conditions for the synthesis of 2,4-aryl-4-((arylsulfonyl)methyl)-4*H*-benzo[*d*][1,3]oxazines from *N*-(2-vinylphenyl)amides and thiols^a^

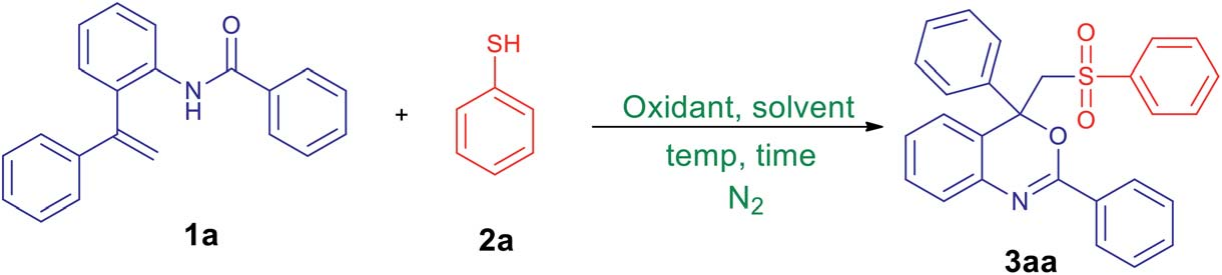						
Entry	Oxidant^b^ (equiv.)	Solvent^c^ (v/v ml)	Charcoal^d^ (g)	Temp. (°C)	Time (h)	Yield^e^ (%)
1	K_2_S_2_O_8_ (2)	CH_3_CN/H_2_O (6 : 4)	—	80	36	26^f^
2	H_2_O_2_ (2)	CH_3_CN/H_2_O (6 : 4)	—	80	36	<5^f^
3	DTBP(2)	CH_3_CN/H_2_O (6 : 4)	—	80	36	<5^f^
4	Na_2_S_2_O_8_ (2)	CH_3_CN/H_2_O (6 : 4)	—	80	36	17^f^
5	(NH_4_)_2_S_2_O_8_ (2)	CH_3_CN/H_2_O (6 : 4)	—	80	36	11^f^
6	K_2_S_2_O_8_ (2)	Methanol	—	80	36	NR^f^
7	K_2_S_2_O_8_ (2)	Acetonitrile	—	80	36	7^f^
8	K_2_S_2_O_8_ (2)	DMSO	—	80	36	12^f^
9	K_2_S_2_O_8_ (2)	EtOAc-H_2_O (5 : 5)	—	80	36	18^f^
10	K_2_S_2_O_8_ (2)	Water	—	80	36	<10^f^
11	K_2_S_2_O_8_ (3)	CH_3_CN/H_2_O (6 : 4)	—	80	36	34^f^
12	K_2_S_2_O_8_ (3.5)	CH_3_CN/H_2_O (6 : 4)	—	80	36	37^f^
13	K_2_S_2_O_8_ (4)	CH_3_CN/H_2_O (6 : 4)	—	80	36	43^f^
14	K_2_S_2_O_8_ (4.5)	CH_3_CN/H_2_O (6 : 4)	—	80	36	45^f^
15	K_2_S_2_O_8_ (5)	CH_3_CN/H_2_O (6 : 4)	—	80	36	44^f^
16	K_2_S_2_O_8_ (4)	CH_3_CN/H_2_O (6 : 4)	—	80	36	54
17	K_2_S_2_O_8_ (4)	CH_3_CN/H_2_O (6 : 4)	—	80	36	50^g^
18	K_2_S_2_O_8_ (4)	CH_3_CN/H_2_O (6 : 4)	—	80	36	31^h^
19	K_2_S_2_O_8_ (4)	CH_3_CN/H_2_O (6 : 4)	—	80	36	22^i^
20	K_2_S_2_O_8_ (4)	CH_3_CN/H_2_O (6 : 4)	0.04	30	52	64
21	K_2_S_2_O_8_ (4)	CH_3_CN/H_2_O (6 : 4)	0.01	40	52	41
22	K_2_S_2_O_8_ (4)	CH_3_CN/H_2_O (6 : 4)	0.02	40	30	47
23	K_2_S_2_O_8_ (4)	CH_3_CN/H_2_O (6 : 4)	0.03	40	15	79
**24**	**K**_**2**_ **S**_**2**_ **O**_**8**_ **(4)**	**CH**_**3**_ **CN/H**_**2**_ **O (6 : 4)**	**0.03**	**50**	**6**	**93**
25	K_2_S_2_O_8_ (4)	CH_3_CN/H_2_O (6 : 4)	0.03	60	6	87
26	K_2_S_2_O_8_ (0)	CH_3_CN/H_2_O (6 : 4)	0.03	50	6	NR^j^

a Unless stated otherwise, all reactions were performed in a Schlenk tube with *N*-(2-(1-phenylvinyl)phenyl)benzamide (1a, 1.0 mmol), benzenethiol (2a, 3.0 mmol) and K_2_S_2_O_8_-activated charcoal in solvent at elevated temperature under nitrogen gas atmosphere.

b Used as received from commercial source.

c Distilled prior to use.

d Obtained from commercial source, *cf.* ESI.

e Isolated yields.

f 2.0 equiv. of benzenethiol used.

g 4.0 equiv. of benzenethiol used.

h Reaction open to air.

i Reaction performed under O_2_ atmosphere.

j Thiol got dimerized into disulfide. NR; no reaction. DTBP; di-*tert*-butyl peroxide.

Recently, granulated activated carbon has been reported to successfully activate persulfate under a mild condition.^13^ It has a certain advantage in being non-metallic species free from metal leaching problems. Also, the activation of persulfate by granulated activated carbon proceeds on the surface of activated carbon during the radical propagation mechanism.^14^ Thus, the influence of activated charcoal was studied for the formation of 3aa from 1a and 2a under the reaction conditions mentioned in entry 16 of Table 1. By addition of 40 mg of activated charcoal to the mixture of 1a, 2a and K_2_S_2_O_8_ in CH_3_CN/H_2_O (6 : 4, v/v mL) at room temperature, expected product 3aa was obtained in 64% yield (Table 1, entry 20). However, rate of the reaction was too low (52 h). More examinations revealed that the complete conversion of mixture of 1a (1.0 equiv.) and 2a (3.0 equiv.) to 3aa in 93% yield required K_2_S_2_O_8_ (4.0 equiv.) and activated charcoal (0.03 g mmol^−1^ of 1a) in CH_3_CN/H_2_O (6 : 4, v/v mL) at 50 °C for 6 h (Table 1, entry 24). Control experiments revealed that K_2_S_2_O_8_ (Table 1, entry 26) was essential, and no desired product was detected in its absence. From these experiments, we determined the optimized conditions as: *N*-(2-vinylphenyl)amide (1.0 equiv.), thiol (3.0 equiv.), K_2_S_2_O_8_ (4.0 equiv.), and activated charcoal (0.03 g mmol^−1^ of *N*-(2-vinylphenyl)amide) in CH_3_CN/H_2_O (6 : 4, v/v mL) under nitrogen gas atmosphere at 50 °C for 6 h (Table 1, entry 24).

With the optimized conditions in hand, the substrate scope and functional group tolerance was studied and the obtained results are presented in Table 2. First, the reactivity of *N*-(2-vinylphenyl)amides with substituent on the benzamide ring (1a–1i) was studied. Indeed, this protocol was found applicable to both electron-donating group and electron-withdrawing group substituent. For instances, methyl-, methoxy-, fluoro-, chloro- and bromo-substituted *N*-(2-vinylphenyl)amides provided the corresponding desired products (3ba–3fa) in 84–92% yields. Notably, the amides with alkyl substituent, such as methyl and *tert*-butyl groups were effective for this reaction and converted to the corresponding benzo[*d*][1,3]oxazines (3ga and 3ha) in moderate to good yields. Nevertheless, no desired product (3ia) could be collected when benzyl-substituted *N*-(2-vinylphenyl)amide was treated with benzenethiol, *cf.*Table 2. Next, we studied the scope of substituted alkenes (1j–1p) in the reaction system. Various substituent include tolyl-, 4-fluorophenyl and 4-chlorophenyl at the α-position of styrenes, afforded products (3ja–3la) in good yields. Likewise, the present system could also be employed to *N*-(2-(prop-1-en-2-yl)phenyl)benzamide and *N*-(2-(prop-1-en-2-yl)phenyl)pivalamide giving products (3ma and 3na) with a yield of 89% and 76% respectively. However, no desired product (3oa or 3pa) could be collected when either *N*-(2-(1-(4-methoxyphenyl)vinyl)phenyl)benzamide or mono-substituted alkene such as *N*-(2-vinylphenyl)benzamide was treated with benzenethiol, *cf.*Table 2.

**Table 2:** Substrate scope for the synthesis of 2,4-aryl-4-((arylsulfonyl)methyl)-4*H*-benzo[*d*][1,3]oxazines from various *N*-(2-vinylphenyl)amides and benzenethiol^a^

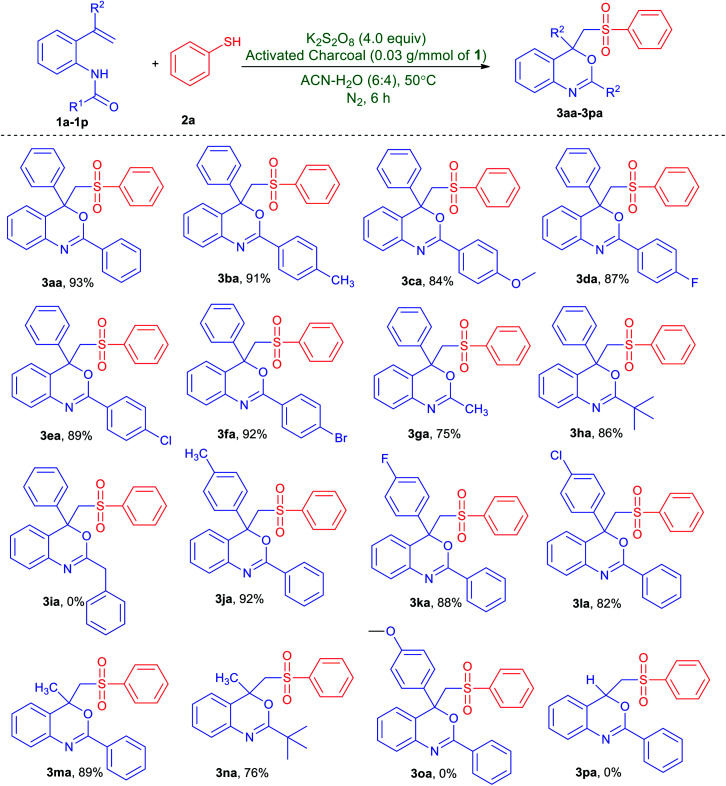

a Unless stated otherwise, all reactions were performed in a Schlenk tube with *N*-(2-vinylphenyl)amides (1a–1p, 1.0 mmol), benzenethiol (2a, 3.0 mmol), K_2_S_2_O_8_ (4.0 mmol) and activated charcoal (0.03 g) in CH_3_CN/H_2_O (6 : 4, v/v mL) under nitrogen gas atmosphere at 50 °C for 6 h.

To further explore the substrate scope, we then studied the scope of thiols (Table 3). Thiols with methyl-, methoxy- and chloro-group at the *para*-position of the arene ring produced the desired products in excellent yields (3ab–3ad). However, 4-nitrobenzenethiol leads to a dramatic decrease of the reaction efficiency and afforded product 3ae in 10% yield. To our delight, this protocol is also applicable to cyclohexanethiol and thiophene-2-thiol and was converted to the corresponding products (3af and 3ag) in significant yield. Unfortunately, thiols with a hydroxy or an amino group on the arene ring could not afford the desired product (3ah and 3ai).

**Table 3:** Substrate scope for the synthesis of 2,4-aryl-4-((arylsulfonyl)methyl)-4*H*-benzo[*d*][1,3]oxazines from *N*-(2-(1-phenylvinyl)phenyl)benzamide and various thiols^a^

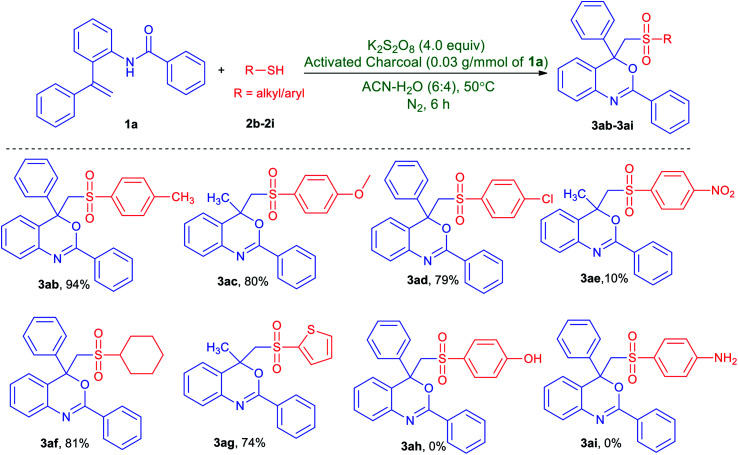

a Unless stated otherwise, all reactions were performed in a Schlenk tube with *N*-(2-(1-phenylvinyl)phenyl)benzamide (1a, 1.0 mmol), thiols (2b–2i, 3.0 mmol), K_2_S_2_O_8_ (4.0 mmol) and activated charcoal (0.03 g) in CH_3_CN/H_2_O (6 : 4, v/v mL) under nitrogen gas atmosphere at 50 °C for 6 h.

**Scheme 3:**
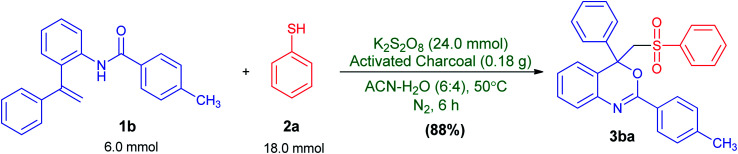
Gram-scale synthesis.

This reaction could also be performed on a gram scale. As shown in Scheme 3, treatment of 1.9 g (6 mmol) of 4-methyl-*N*-(2-(1-phenylvinyl)phenyl)benzamide (1b) with 3 equiv. of benzenethiol (2a) under the optimized reaction conditions (Table 1, entry 24) afforded the desired 4-phenyl-4-((phenylsulfonyl)methyl)-2-*p*-tolyl-4*H*-benzo[*d*][1,3]oxazine (3ba) in 88% (2.4 g) isolated yield, clearly demonstrating the preparative practicality of this protocol.

**Scheme 4:**
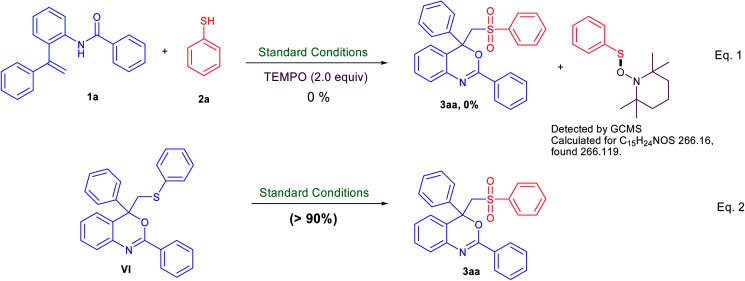
Control experiments for mechanistic studies.

To understand the mechanism of this transformation, some control experiments were carried out as described in Scheme 4. On addition of 2 equiv. of radical scavenger 2,2,6,6-tetramethylpiperidine-1-oxyl (TEMPO) under the standard conditions, no desired product (3aa) was observed (instead thiol·TEMPO adduct detected by GCMS analysis). In other words, TEMPO completely inhibited this reaction indicating that the reaction follows a radical pathway.^7,8^ In addition, the sulfur-containing benzo[*d*][1,3]oxazine (VI) could also be converted to the sulfone-containing benzo[*d*][1,3]oxazine (3aa) in good yield under similar reaction conditions in the absence of thiol. Thus, it would be reasonable to deduce that sulfur-containing benzo[*d*][1,3]oxazine (VI) was the plausible intermediate in this reaction.^6^

**Scheme 5:**
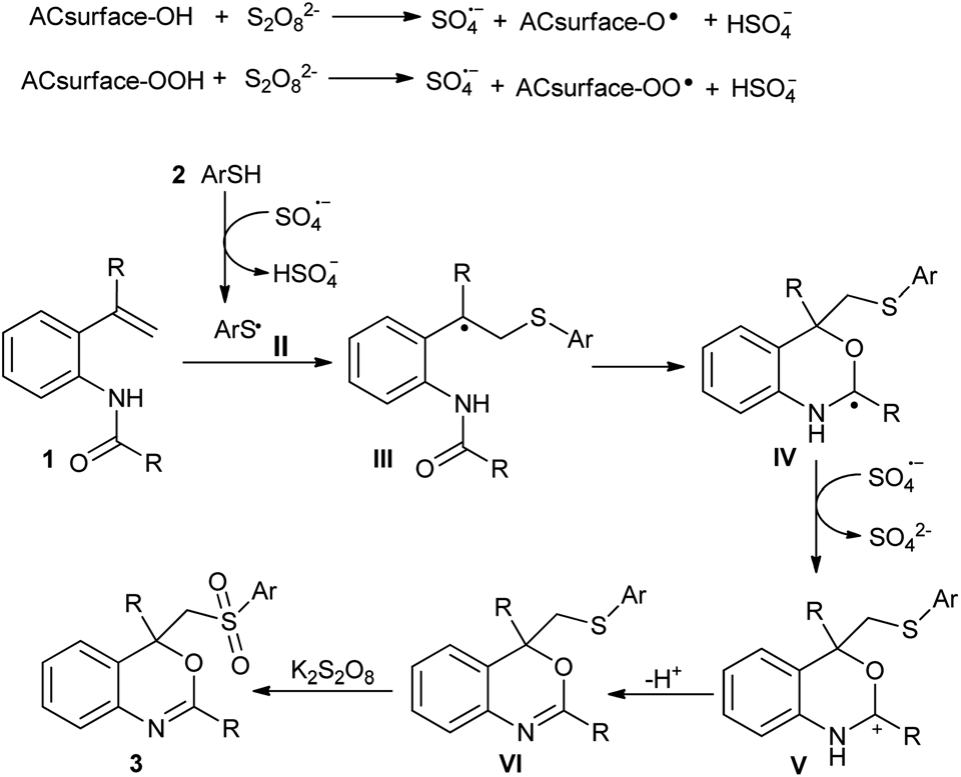
A plausible mechanism for the formation of 2,4-aryl-4-((arylsulfonyl)methyl)-4*H*-benzo[*d*][1,3]oxazines from *N*-(2-vinylphenyl)amides and thiols.

On the basis of the results described above and previous reports, a plausible mechanism is outlined in Scheme 5. Initially, the activated charcoal-assisted heterolytic cleavage of S_2_O_8_^2−^ affords sulfate radical anions (SO_4_˙^−^), which then abstract the hydrogen atoms from the thiol (2) to afford a sulfur-centered sulfonyl radical (II).^13,14^ The addition of radical II to C=C bond of *N*-(2-vinylphenyl)amide (1) would lead to the formation of alkyl radical III. Later, III undergoes an intramolecular radical cyclization to provide a new radical intermediate IV. Subsequently, the radical IV was further oxidized to the corresponding carbocation (V) by oxidant followed by deprotonation to afford the sulfur-containing benzo[*d*][1,3]oxazine (VI).^5^ However, a cationic cyclization cannot be excluded completely, in which the alkyl radical intermediate III is further oxidized to carbocation and subsequently trapped by the carbonyl group of amide.^6^ The resulting VI would be rapidly oxidized to desired sulfone-containing benzo[*d*][1,3]oxazine (3) by K_2_S_2_O_8_.^7,13^ The influence of activated charcoal on reactivity of K_2_S_2_O_8_ is not clear, however, we believe that the high surface area and micro porosity of activated charcoal may play role through widespread interactions and scission of S_2_O_8_^2−^ to more powerful sulfate radical anions (SO_4_˙^−^) that accelerates the reaction under mild conditions.^14^

In summary, a mild and cost-efficient protocol was developed for the synthesis of 2,4-aryl-4-((arylsulfonyl)methyl)-4*H*-benzo[*d*][1,3]oxazines from *N*-(2-vinylphenyl)amides and thiols by employing mixture of K_2_S_2_O_8_-activated charcoal in aqueous acetonitrile solution at 50 °C. The facile formation of new C–S, C–O and S–O bonds take place in a one-pot procedure. Versatility of this synthetic method for a broad range of *N*-(2-vinylphenyl)amides and thiols as well as the benefits of use of easily accessible K_2_S_2_O_8_-activated charcoal mixture and thiols, respectively, as an oxidant and sulfonylating precursors. Further studies on the mechanism and applications are ongoing in our laboratory.

## Experimental section

### General procedure for the synthesis of 2,4-aryl-4-((arylsulfonyl)methyl)-4*H*-benzo[*d*][1,3]oxazines


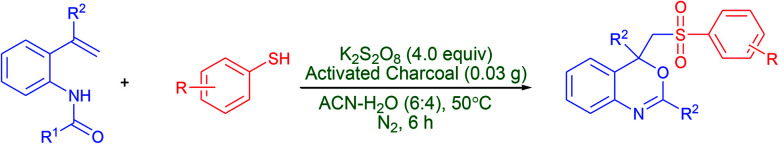
An oven-dried Schlenk-tube equipped with a magnetic stir bar was charged with *N*-(2-vinylphenyl)amides (1.0 mmol, 1.0 equiv.), thiol (3.0 mmol, 3.0 equiv.), K_2_S_2_O_8_ (4.0 mmol, 4.0 equiv.) and activated charcoal (0.03 g). To this mixture, CH_3_CN/H_2_O (6 : 4, v/v mL, 10 mL) was added. Then, the tube was sealed and inlet/outlet for N_2_ gas was provided by a side-neck. Resultant mixture was vigorously stirred under nitrogen gas atmosphere at 50 °C for 6 h. After the completion (as indicated by TLC, ≈6 h) volatiles were evaporated under reduced pressure and then admixed with aqueous K_2_CO_3_ solution (20 mL). The organic matters are extracted with ethyl acetate, dried over Na_2_SO_4_ and evaporated under reduced pressure to yield a pale-yellow gummy-solid, which was purified by a column chromatography using a mixture of ethyl acetate and hexane. The identity and purity of the product was confirmed by spectroscopic analysis as well as by a comparison with authentic samples spectra, *vide infra*.

## Conflicts of interest

There are no conflicts to declare.

## Supplementary Material

RA-011-D1RA02377B-s001
