# Supplementary material for: Persulfate-activated charcoal mixture: an efficient oxidant for the synthesis of sulfonated benzo[d][1,3]oxazines from N-(2-vinylphenyl)amides and thiols in aqueous solution
Source: RSC Adv. 2021 Apr 27;11(26):15573–80. doi: 10.1039/d1ra02377b (PMC9029395; doi:10.1039/d1ra02377b)
Supplement: RA-011-D1RA02377B-s001 [file RA-011-D1RA02377B-s001.pdf]

Electronic Supplementary Material (ESI) for RSC Advances.
This journal is © The Royal Society of Chemistry 2021

Supplementary Information

Persulfate-activated charcoal mixture: an efficient oxidant for the synthesis of sulfonated benzo[d][1,3]oxazines from N-(2-vinylphenyl)amides and thiols in aqueous solution

Palani Natarajan*, Priya and Deachen Chuskit
Department of Chemistry & Centre for Advanced Studies in Chemistry, Panjab University, Chandigarh - 160 014, India
pnataraj@pu.ac.in

TABLE OF CONTENTS

1. General Information S2
2. General procedure for the synthesis of products S2-S3
3. Experimental characterization data for products S4-S13
4. References S13
5. Copies of ^{1}H and ^{13}C NMR spectra of products S14-S32

General Information

Unless otherwise stated, all reagents and substrates were purchased from commercial sources with the best quality and they were used without further purification. All solvents were distilled according to the established procedures[1] and de-aerated prior to use. Activated charcoal was obtained from Merck Company (catalogue number 102186) and dried at 60 °C under vacuum. The column chromatography was performed using silica gel with 100-200 mesh size. Reactions were monitored by analytical thin layer chromatography on silica gel and visualization was accomplished by irradiation with short wave UV light at 254 nm and near UV 366 nm lights. All reactions were carried out under nitrogen gas atmosphere using standard Schlenk-tube. All products are known and were characterized by their ^{1}H NMR, and ^{13}C NMR followed by a comparison with authentic samples spectra. Chemical shifts are expressed as δ-value in parts per million (ppm) and were calibrated using the residual protinated solvent as an internal standard. The peak patterns are indicated as follows: s, singlet; d, doublet; t, triplet; m, multiplet; q, quartet and so on. The coupling constants, J, are reported in Hertz (Hz). High resolution mass spectra were collected by positive mode electrospray ionization (ESI) using Waters-Q-TOF-Premier mass spectrometer.

Experimental Section

General procedure for the synthesis of 2,4-aryl-4-((arylsulfonyl)methyl)-4H-benzo[d][1,3]oxazines

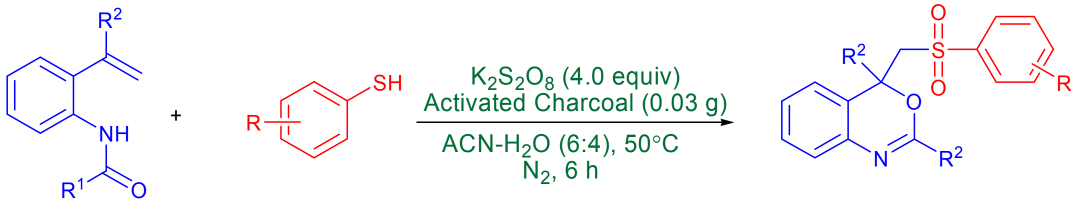

An oven-dried Schlenk-tube equipped with a magnetic stir bar was charged with *N*-(2-vinylphenyl)amides (1.0 mmol, 1.0 equiv.), thiol (3.0 mmol, 3.0 equiv.), $K_2S_2O_8$ (4.0 mmol, 4.0 equiv.) and activated charcoal (0.03 g). To this mixture, CH_3CN/H_2O (6:4, v/v mL, 10 mL) was added. Then, the tube was sealed and inlet/outlet for N_2 gas was provided by a side-neck. Resultant mixture was vigorously stirred under nitrogen gas atmosphere at 50 °C for 6 h. After the completion (as indicated by TLC, ≈6 h) volatiles were evaporated under reduced pressure and then admixed with aqueous K_2CO_3 solution (20 mL). The organic matters are extracted with

ethyl acetate, dried over Na_2SO_4 and evaporated under reduced pressure to yield a pale-yellow gummy-solid, which was purified by a column chromatography using a mixture of ethyl acetate and hexane. The identity and purity of the product was confirmed by spectroscopic analysis as well as by a comparison with authentic samples spectra, vide infra.

Experimental characterization data for products

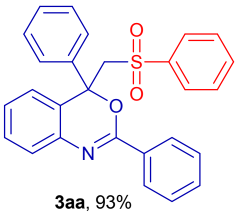

3aa, 93%

2,4-diphenyl-4-(phenylsulfonyl)methyl)-4H-benzo[d][1,3]oxazine (3aa):[2] By following the typical procedure, the product (**3aa**) was isolated as a white solid, 410 mg (93% yield), from a column chromatography (petroleum ether/ethyl acetate = 7/3)), mp.128-129 °C. IR (KBr, cm^{-1}): 3062, 2923, 2853, 1623, 1575, 1482, 1320, 1301, 1272, 1078. ^{1}H NMR (300 MHz, CD_2Cl_2): δ 7.84 (d, J = 7.8 Hz, 2H), 7.61 (d, J = 7.8 Hz, 2H), 7.39 (t, J = 7.4 Hz, 1H), 7.33-7.26 (m, 4H), 7.24-7.17 (m, 5H), 7.14 (s, 5H), 4.22 (q, J = 15.6 Hz, 2H). ^{13}C NMR (75 MHz, CD_2Cl_2): δ 154.6, 141.7, 140.8, 138.7, 133.2, 131.7, 131.6, 129.6, 128.9, 128.8, 128.6, 128.2, 128.2, 127.9, 126.3, 126.0, 125.2, 124.1, 80.8, 64.5. HRMS (ESI, m/z): Calculated for $C_{27}H_{22}NO_3S$ $(M+H)^+$ 440.1315, found 440.1309.

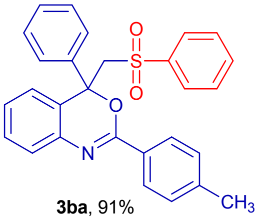

3ba, 91%

4-phenyl-4-((phenylsulfonyl)methyl)-2-(p-tolyl)-4H-benzo[d][1,3]oxazine (3ba):[2] By following the typical procedure, the product (**3ba**) was isolated as a white solid, 415 mg (91% yield), from a column chromatography (petroleum ether/ethyl acetate = 7/3), mp 155-156 °C. IR (KBr, cm^{-1}): 3054, 2938, 2836, 1625, 1577, 1488, 1319, 1297, 1281, 1265, 1081. ^{1}H NMR (300 MHz, CD_2Cl_2): δ 7.82 (d, J = 7.4 Hz, 2H), 7.66 (d, J = 5.2 Hz, 2H), 7.42 (s, 1H), 7.34 (s, 1H), 7.31-7.26 (m, 4H), 7.21 (s, 6H), 7.18 (d, J = 7.4 Hz, 2H), 4.28 (q, J = 15.1 Hz, 2H), 2.40 (s, 3H). ^{13}C NMR (75 MHz, CD_2Cl_2): δ 154.8, 142.1, 141.6, 140.7, 138.9, 133.3, 129.6, 128.9, 128.7, 128.6, 128.4, 128.2, 127.9, 126.2, 125.8, 125.3, 125.1, 124.3, 80.8, 64.5, 21.6. HRMS (ESI, m/z): Calculated for $C_{28}H_{24}NO_3S$ $(M+H)^+$ 454.1471, found 454.1457.

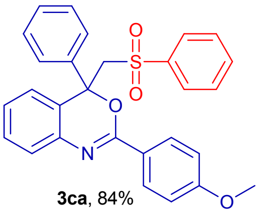

3ca, 84%

2-(4-methoxyphenyl)-4-phenyl-4-((phenylsulfonyl)methyl)-4H-benzo[d][1,3]oxazine (3ca):[2] By following the typical procedure, the product (**3ca**) was isolated as a white solid, 395 mg (84% yield), from a column chromatography (petroleum ether/ethyl acetate = 7/3), mp 200-202 °C. IR (KBr, cm^{-1}): 3057, 2934, 2838, 1580, 1486, 1317, 1302, 1287, 1079. ^{1}H NMR (300 MHz, CD_2Cl_2): δ 7.91-7.88 (m, 2H), 7.69-7.66 (m, 2H), 7.42 (t, *J* = 7.2 Hz, 1H), 7.33 (td, *J* = 7.6, 1.2 Hz, 1H), 7.30 (t, *J* = 7.6 Hz, 3H), 7.25-7.23 (m, 1H), 7.23-7.18 (m, 6H), 6.90-6.87 (m, 2H), 4.31-4.27 (m, 2H), 3.88 (s, 3H). ^{13}C NMR (75 MHz, CD_2Cl_2): δ 162.4, 154.7, 141.7, 140.8, 139.1, 133.2, 129.8, 129.7, 128.9, 128.6, 128.6, 128.2, 125.9, 125.5, 125.3, 125.2, 124.1, 124.0, 113.6, 80.8, 64.6, 55.4. HRMS (ESI, m/z): Calculated for $C_{28}H_{24}NO_4S$ $(M+H)^+$ 470.1421, found 470.1415.

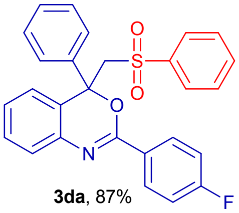

3da, 87%

2-(4-fluorophenyl)-4-phenyl-4-((phenylsulfonyl)methyl)-4H-benzo[d][1,3]oxazine (3da):[2] By following the typical procedure, the product (**3da**) was isolated as a pale yellow solid, 400 mg (87% yield), from a column chromatography (petroleum ether/ethyl acetate = 7/3), mp 146-147 °C. IR (KBr, cm^{-1}): 3061, 2985, 2842, 1634, 1448, 1377, 1322, 1290, 1244, 1048. ^{1}H NMR (300 MHz, CD_2Cl_2): δ 7.81-7.78 (m, 2H), 7.66 (dd, *J* = 8.2, 1.2 Hz, 2H), 7.53-7.51 (m, 1H), 7.44 (t, *J* = 7.4 Hz, 1H), 7.36 (td, *J* = 7.6, 1.8 Hz, 2H), 7.32 (t, *J* = 7.6 Hz, 2H), 7.30-7.27 (m, 2H), 7.26-7.21 (m, 4H), 7.07-7.05 (m, 2H), 4.28 (q, J = 15.4 Hz, 2H). ^{13}C NMR (75 MHz, CD_2Cl_2): δ 165.7, 164.2, 153.8, 141.4, 140.8, 138.6, 133.4, 130.2, 130.1, 129.8, 128.9, 128.7, 128.2, 127.8, 126.5, 125.9, 125.3, 125.1, 124.1, 115.4, 115.2, 81.1, 64.4. HRMS (ESI, m/z): Calculated for $C_{27}H_{21}FNO_3S$ $(M+H)^+$ 458.1221, found 458.1217.

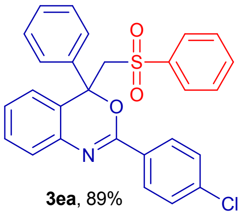

3ea, 89%

2-(4-chlorophenyl)-4-phenyl-4-((phenylsulfonyl)methyl)-4H-benzo[d][1,3]oxazine (3ea):[2] By following the typical procedure, the product (**3ea**) was isolated as a white solid, 423 mg (89% yield), from a column chromatography (petroleum ether/ethyl acetate = 7/3), mp 150-151 °C. IR (KBr, cm^{-1}): 3062, 2927, 2850, 1620, 1576, 1475, 1316, 1301, 1287, 1258, 1086. ^1H NMR (300 MHz, CD_2Cl_2): δ 7.89-7.87 (m, 2H), 7.68-7.66 (m, 2H), 7.44 (t, J = 7.4 Hz, 1H), 7.38-7.34 (m, 3H), 7.31-7.27 (m, 4H), 7.25-7.19 (m, 6H), 4.28 (q, J = 15.6 Hz, 2H). ^{13}C NMR (75 MHz, CD_2Cl_2): δ 153.9, 141.4, 140.8, 138.6, 137.8, 133.3, 130.1, 129.7, 129.2, 129.1, 128.7, 128.5, 128.2, 126.6, 126.1, 125.2, 124.1, 81.2, 64.5. HRMS (ESI, m/z): Calculated for $C_{27}H_{21}ClNO_3S$ $(M+H)^+$ 474.0925, found 474.0916.

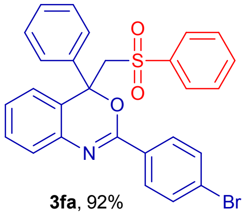

3fa, 92%

2-(4-bromophenyl)-4-phenyl-4-((phenylsulfonyl)methyl)-4H-benzo[d][1,3]oxazine (3fa):[2] By following the typical procedure, the product (**3fa**) was isolated as a white solid, 480 mg (92% yield), from a column chromatography (petroleum ether/ethyl acetate = 7/3), mp 135-136 °C. IR (KBr, cm^{-1}): 3060, 2927, 2853, 1620, 1578, 1473, 1317, 1294, 1262, 1081. ^1H NMR (300 MHz, CD_2Cl_2): δ 7.81 (d, J = 8.2 Hz, 2H), 7.68 (d, J = 7.8 Hz, 2H), 7.50 (d, J = 8.2 Hz, 2H), 7.42 (t, J = 7.4 Hz, 1H), 7.38-7.34 (m, 1H), 7.31 (t, J = 7.8 Hz, 2H), 7.27-7.24 (m, 3H), 7.22 (d, J = 7.4 Hz, 3H), 7.21-7.18 (m, 2H), 4.27 (q, J = 15.6 Hz, 2H). ^{13}C NMR (75 MHz, CD_2Cl_2): δ 153.8, 141.4, 140.7, 138.4, 133.3, 131.5, 130.6, 129.7, 129.4, 129.1, 128.7, 128.2, 126.6, 126.4, 126.1, 125.2, 125.1, 124.2, 81.2, 64.4. HRMS (ESI, m/z): Calculated for $C_{27}H_{21}BrNO_3S$ $(M+H)^+$ 518.0420, found 518.0413.

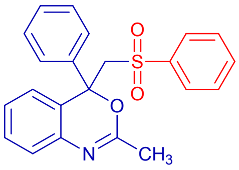

3ga, 75%

2-methyl-4-phenyl-4-((phenylsulfonyl)methyl)-4H-benzo[d][1,3]oxazine (3ga):[4] By following the typical procedure, the product (**3ga**) was isolated as a pale yellow oil, 285 mg (75% yield), from a column chromatography (petroleum ether/ethyl acetate = 7.5/2.5). IR (KBr, cm^{-1}): 3059, 2930, 2839, 1647, 1583, 1488, 1423, 1324, 1291, 1259. ^{1}H NMR (300 MHz, CD_2Cl_2): δ 7.67 (d, J = 7.6 Hz, 2H), 7.56 (t, J = 7.6 Hz, 1H), 7.41 (t, J = 7.6 Hz, 2H), 7.32-7.26 (m, 4H), 7.23-7.21 (m, 2H), 7.14-7.08 (m, 2H), 7.05-7.03 (m, 1H), 4.13 (q, J = 15.4 Hz, 2H), 1.96 (s, 3H). ^{13}C NMR (75 MHz, CD_2Cl_2): δ 158.3, 141.6, 140.6, 138.1, 133.5, 129.6, 128.9, 128.7, 128.3, 126.2, 125.6, 125.4, 125.1, 123.4, 80.5, 64.2, 21.3. HRMS (ESI, m/z): Calculated for $C_{22}H_{20}NO_3S$ $(M+H)^+$ 378.1158, found 378.1149.

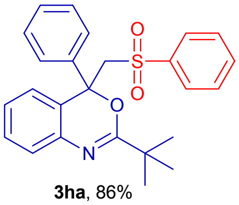

3ha, 86%

2-(*tert*-butyl)-4-phenyl-4-((phenylsulfonyl)methyl)-4H-benzo[d][1,3]oxazine (3ha):[4] By following the typical procedure, the product (**3ha**) was isolated as a yellow oil, 365 mg (86% yield), from a column chromatography (petroleum ether/ethyl acetate = 8/2). IR (KBr, cm^{-1}): 3063, 2959, 2856, 1625, 1579, 1488, 1318, 1289, 1269, 1075. ^{1}H NMR (300 MHz, CD_2Cl_2): δ 7.65-6.58 (m, 2H), 7.53-7.50 (m, 1H), 7.38 (dd, J = 8.4, 7.6 Hz, 2H), 7.28-7.24 (m, 4H), 7.19-7.16 (m, 3H), 7.02-6.97 (m, 1H), 6.94 (d, J = 1.2 Hz, 1H), 4.28 (d, J = 15.2 Hz, 1H), 4.21 (d, J = 15.2 Hz, 1H), 1.15 (s, 9H). ^{13}C NMR (75 MHz, CD_2Cl_2): 166.2, 142.5, 140.8, 139.0, 133.4, 129.4, 129.1, 128.7, 128.4, 128.2, 126.0, 125.7, 125.6, 125.5, 122.4, 80.2, 64.9, 37.3, 27.5. HRMS (ESI, m/z): Calculated for $C_{25}H_{26}NO_3S$ $(M+H)^+$ 420.1628, found 420.1617.

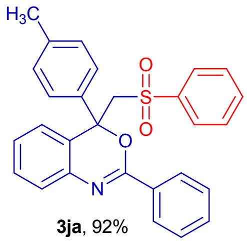

3ja, 92%

2-phenyl-4-((phenylsulfonyl)methyl)-4-(*p*-tolyl)-4H-benzo[d][1,3]oxazine (3ja):[2] By following the typical procedure, the product (**3ja**) was isolated as a white solid, 420 mg (92% yield), from a column chromatography (petroleum ether/ethyl acetate = 7/3), mp 156-157 °C. IR (KBr, cm^{-1}): 3061, 2925, 2838, 1626, 1579, 1488, 1319, 1287, 1276, 1076. ^{1}H NMR (300 MHz, CD_2Cl_2): δ 7.92 (d, *J* = 6.4 Hz, 2H), 7.68 (d, *J* = 6.4 Hz, 2H), 7.47 (s, 1H), 7.38-7.34 (m, 4H), 7.26 (s, 4H), 7.24 (d, *J* = 6.4 Hz, 1H), 7.12 (d, *J* = 7.2 Hz, 2H), 7.01 (d, *J* = 7.2 Hz, 2H), 4.33-4.24 (m, 2H), 2.23 (s, 3H). ^{13}C NMR (75 MHz, CD_2Cl_2): δ 154.6, 140.8, 138.7, 138.6, 138.5, 133.2, 131.8, 131.5, 129.5, 129.3, 128.7, 128.3, 128.1, 127.8, 126.4, 125.9, 125.3, 124.2, 80.9, 64.6, 21.0. HRMS (ESI, m/z): Calculated for $C_{28}H_{24}NO_3S$ $(M+H)^+$ 454.1471, found 454.1459.

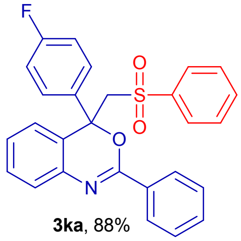

3ka, 88%

4-(4-fluorophenyl)-2-phenyl-4-((phenylsulfonyl)methyl)-4H-benzo[d][1,3]oxazine (3ka):[2] By following the typical procedure, the product (**3ka**) was isolated as a pale yellow solid, 405 mg (88% yield), from a column chromatography (petroleum ether/ethyl acetate = 7/3), mp 141-142 °C. IR (KBr, cm^{-1}): 3056, 2931, 2839, 1627, 1581, 1496, 1317, 1266, 1074. ^{1}H NMR (300 MHz, CD_2Cl_2): δ 7.96-7.93 (m, 2H), 7.68-7.65 (m, 2H), 7.47 (t, *J* = 7.6 Hz, 1H), 7.42 (t, *J* = 7.4 Hz, 1H), 7.39-7.34 (m, 3H), 7.33-7.26 (m, 4H), 7.25-7.19 (m, 3H), 6.93-6.89 (m, 2H), 4.29 (q, *J* = 15.6 Hz, 2H). ^{13}C NMR (75 MHz, CD_2Cl_2): δ 162.6 (d, *J* = 247.2 Hz), 154.7, 140.6, 138.7, 137.3 (d, *J* = 3.1 Hz), 133.2, 131.6, 131.4, 129.8, 128.9, 128.3, 128.1, 127.9, 127.4 (d, *J* = 8.2 Hz), 126.4, 126.1, 125.0, 124.2, 115.6 (d, *J* = 21.6 Hz), 80.4, 64.5. HRMS (ESI, m/z): Calculated for $C_{27}H_{21}FNO_3S$ $(M+H)^+$ 458.1221, found 458.1217.

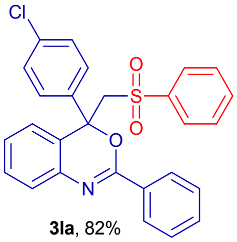

3la, 82%

4-(4-chlorophenyl)-2-phenyl-4-((phenylsulfonyl)methyl)-4H-benzo[d][1,3]oxazine (3la):[2] By following the typical procedure, the product (**3la**) was isolated as a white solid, 390 mg (82% yield), from a column chromatography (petroleum ether/ethyl acetate = 7/3), mp 155-156 °C. IR (KBr, cm^{-1}): 3055, 2932, 2839, 1628, 1582, 1496, 1313, 1267, 1076. ^{1}H NMR (300 MHz, CD_2Cl_2): δ 7.96-7.94 (m, 2H), 7.71-7.65 (m, 2H), 7.49 (d, *J* = 7.6 Hz, 1H), 7.42-7.35 (m, 4H), 7.31-7.27 (m, 3H), 7.26-7.21 (m, 2H), 7.20-7.14 (m, 4H), 4.26 (q, *J* = 15.6 Hz, 2H). ^{13}C NMR (75 MHz, CD_2Cl_2): δ 154.7, 140.7, 139.8, 138.6, 134.7, 133.2, 131.7, 131.4, 129.8, 129.1, 128.8, 128.3, 128.1, 127.8, 126.9, 126.5, 126.1, 124.9, 124.0, 80.4, 64.1. HRMS (ESI, m/z): Calculated for $C_{27}H_{21}ClNO_3S$ $(M+H)^+$ 474.0925, found 474.0917.

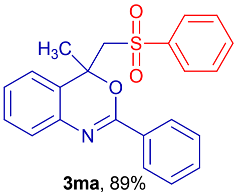

3ma, 89%

4-methyl-2-phenyl-4-((phenylsulfonyl)methyl)-4H-benzo[d][1,3]oxazine (3ma):[3] By following the typical procedure, the product (**3ma**) was isolated as a colorless oil, 340 mg (89% yield), from a column chromatography (petroleum ether/ethyl acetate = 8/2). IR (KBr, cm^{-1}): 3060, 2931, 2839, 1648, 1583, 1489, 1424, 1322, 1291, 1260. ^{1}H NMR (300 MHz, CD_2Cl_2): δ 8.07-8.04 (m, 2H), 7.73 (dd, *J* = 8.2, 0.6 Hz, 2H), 7.51-7.46 (m, 2H), 7.42 (t, *J* = 7.8 Hz, 2H), 7.36 (t, *J* = 8.2 Hz, 2H), 7.31-7.28 (m, 1H), 7.24 (d, *J* = 6.0 Hz, 1H), 7.19-7.16 (m, 1H), 7.14-7.11 (m, 1H), 3.82 (d, *J* = 14.8 Hz, 1H), 3.64 (d, *J* = 14.8 Hz, 1H), 2.10 (s, 3H). ^{13}C NMR (75 MHz, CD_2Cl_2): δ 155.7, 140.6, 138.2, 133.4, 132.1, 131.5, 129.6, 129.1, 128.2, 128.1, 127.6, 127.0, 125.7, 123.2, 77.8, 64.0, 27.1. HRMS (ESI, m/z): Calculated for $C_{22}H_{20}NO_3S$ $(M+H)^+$ 378.1158, found 378.1149.

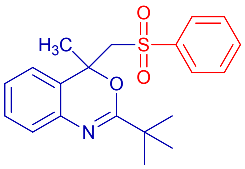

3na, 76%

(*tert*-butyl)-4-methyl-4-((phenylsulfonyl)methyl)-4*H*-benzo[d][1,3]oxazine (3na):[4] By following the typical procedure, the product (**3na**) was isolated as a pale yellow oil, 290 mg (76% yield), from a column chromatography (petroleum ether/ethyl acetate = 9/1). IR (KBr, cm^{-1}): 3059, 2929, 2839, 1646, 1582, 1489, 1423, 1221, 1288, 1262, 1073. ^{1}H NMR (300 MHz, CD_2Cl_2): δ 7.71-7.67 (m, 2H), 7.53 (d, J = 7.4 Hz, 1H), 7.42 (t, J = 7.8 Hz, 2H), 7.20 (d, J = 6.0 Hz, 1H), 7.11-7.09 (m, 1H), 7.04-6.95 (m, 2H), 3.74 (d, J = 14.8 Hz, 1H), 3.66 (d, J = 14.8 Hz, 1H), 1.89 (s, 3H), 1.23 (s, 9H). ^{13}C NMR (75 MHz, CD_2Cl_2): δ 166.8, 140.7, 138.2, 133.4, 129.2, 129.1, 127.7, 126.4, 126.1, 125.2, 123.3, 77.1, 64.2, 37.2, 27.3. HRMS (ESI) calculated for $C_{20}H_{24}NO_3S$ $[M+H]^+$: 358.1298; found: 358.1289.

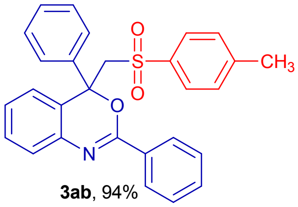

3ab, 94%

2,4-diphenyl-4-(tosylmethyl)-4H-benzo[d][1,3]oxazine (3ab):[2] By following the typical procedure, the product (**3ab**) was isolated as a white solid, 430 mg (94% yield), from a column chromatography (petroleum ether/ethyl acetate = 7/3), mp 152-153 °C. IR (KBr, cm^{-1}): 3055, 2985, 2927, 1628, 1490, 1424, 1318, 1267, 1086. ^{1}H NMR (300 MHz, CD_2Cl_2): δ 7.92-7.88 (m, 2H), 7.54 (d, J = 7.6 Hz, 2H), 7.49-7.46 (m, 1H), 7.39-7.35 (m, 3H), 7.34-7.27 (m, 3H), 7.25 (dd, J = 8.4, 1.8 Hz, 1H), 7.23-7.21 (m, 4H), 7.05 (d, J = 7.8 Hz, 2H), 4.28 (q, J = 15.2 Hz, 2H), 2.22 (s, 3H). ^{13}C NMR (75 MHz, CD_2Cl_2): δ 154.7, 144.3, 141.9, 138.8, 137.8, 131.6, 131.4, 129.5, 129.4, 128.8, 128.6, 128.2, 128.1, 127.8, 126.3, 126.0, 125.5, 125.2, 123.9, 80.8, 64.6, 21.5. HRMS (ESI, m/z): Calculated for $C_{28}H_{24}NO_3S$ $(M+H)^+$ 454.1471, found 454.1462.

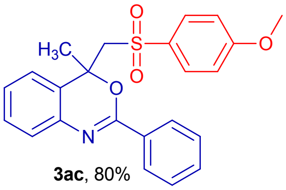

3ac, 80%

4-(((4-methoxyphenyl)sulfonyl)methyl)-4-methyl-2-phenyl-4*H*-benzo[d][1,3]oxazine (3ac):[3] By following the typical procedure, the product (**3ac**) was isolated as a pale yellow oil, 330 mg (80% yield), from a column chromatography (petroleum ether/ethyl acetate = 8/2). IR (KBr, cm^{-1}): 3055, 2962, 2937, 2838, 1627, 1592, 1491, 1326, 1291, 1264, 1032. ^{1}H NMR (300 MHz, CD_2Cl_2): δ 8.02 (d, *J* = 7.2 Hz, 2H), 7.62 (d, *J* = 8.8 Hz, 2H), 7.46 (t, *J* = 7.2 Hz, 1H), 7.39 (t, *J* = 7.2 Hz, 2H), 7.35-7.24 (m, 2H), 7.22-7.11 (m, 2H), 6.76 (d, *J* = 8.8 Hz, 2H), 3.83 (d, *J* = 14.8 Hz, 1H), 3.73 (s, 3H), 3.60 (d, *J* = 14.8 Hz, 1H), 2.05 (s, 3H). ^{13}C NMR (75 MHz, CD_2Cl_2): δ 163.5, 155.4, 138.2, 132.1, 132.0, 131.5, 129.9, 129.4, 128.1, 126.9, 126.8, 125.4, 123.2, 114.3, 77.9, 64.1, 55.6, 27.3. HRMS (ESI, m/z): Calculated for $C_{23}H_{22}NO_4S$ $(M+H)^+$ 408.1264, found 408.1256.

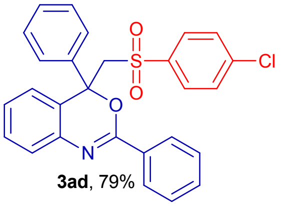

3ad, 79%

4-(((4-chlorophenyl)sulfonyl)methyl)-2,4-diphenyl-4H-benzo[d][1,3]oxazine (3ad):[2] By following the typical procedure, the product (**3ad**) was isolated as a white solid, 375 mg (79% yield), from a column chromatography (petroleum ether/ethyl acetate = 7/3), mp 151-152 °C. IR (KBr, cm^{-1}): 3061, 2926, 2852, 1625, 1577, 1480, 1323, 1290, 1261, 1084. ^{1}H NMR (300 MHz, CD_2Cl_2): δ 7.91-7.88 (m, 2H), 7.58-7.56 (m, 2H), 7.51-7.48 (m, 1H), 7.42-7.39 (m, 2H), 7.36 (dd, *J* = 7.2, 0.8 Hz, 1H), 7.31-7.28 (m, 2H), 7.26-7.24 (m, 1H), 7.23-7.21 (m, 5H), 7.20-7.17 (m, 2H), 4.32-4.28 (m, 2H). ^{13}C NMR (75 MHz, CD_2Cl_2): δ 154.6, 141.7, 140.2, 139.1, 138.7, 131.8, 131.4, 129.7, 129.6, 129.3, 128.7, 128.6, 128.4, 127.6, 126.4, 126.1, 125.3, 125.2, 123.9, 80.8 64.6. HRMS (ESI, m/z): Calculated for $C_{27}H_{21}ClNO_3S$ $(M+H)^+$ 474.0925, found 474.0917.

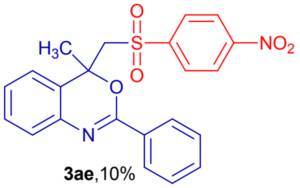

3ae,10%

4-methyl-4-(((4-nitrophenyl)sulfonyl)methyl)-2-phenyl-4*H*-benzo[d][1,3]oxazine (3ae):[3] By following the typical procedure, the product (**3ae**) was isolated as a pale yellow solid, 45 mg (10% yield), from a column chromatography (petroleum ether/ethyl acetate = 7/3), mp 109-110 °C. IR (KBr, cm^{-1}): 3062, 2934, 2839, 1627, 1592, 1487, 1341, 1269, 1082. ^{1}H NMR (300 MHz, CD_2Cl_2): δ 8.05 (d, *J* = 8.0 Hz, 2H), 7.87 (d, *J* = 7.8 Hz, 2H), 7.83 (d, *J* = 8.0 Hz, 2H), 7.46 (t, *J* = 7.2 Hz, 1H), 7.42-7.31 (m, 3H), 7.28 (d, *J* = 5.4 Hz, 1H), 7.19 (t, *J* = 7.2 Hz, 1H), 7.13 (d, *J* = 7.6 Hz, 1H), 3.98 (d, *J* = 15.2 Hz, 1H), 3.79 (d, *J* = 15.2 Hz, 1H), 2.04 (s, 3H). ^{13}C NMR (75 MHz, CD_2Cl_2): δ 154.9, 150.3, 145.4, 138.1, 131.8, 131.6, 129.8, 129.2, 128.5, 128.1, 127.7, 127.2, 125.8, 124.2, 123.3, 77.8, 64.4, 28.2. HRMS (ESI, m/z): Calculated for $C_{22}H_{19}N_2O_5S$ $(M+H)^+$ 423.1009, found 423.1017.

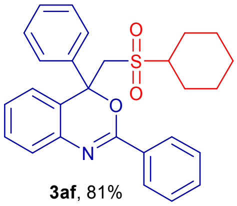

3af, 81%

4-((cyclohexylsulfonyl)methyl)-2,4-diphenyl-4H-benzo[d][1,3]oxazine (3af): By following the typical procedure, the product (**3af**) was isolated as a colorless oil, 410 mg (81% yield), from a column chromatography (petroleum ether/ethyl acetate = 9/1). IR (KBr, cm^{-1}): 3063, 2924, 2852, 1624, 1574, 1483, 1321, 1301, 1274, 1078. ^{1}H NMR (300 MHz, CD_2Cl_2): δ 7.41 (t, *J* = 7.2 Hz, 1H), 7.34-7.27 (m, 4H), 7.24-7.18 (m, 4H), 7.16 (s, 5H), 4.27 (q, *J* = 15.2 Hz, 2H), 2.57-2.49 (m, 1H), 2.11-2.00 (m, 2H), 1.92-1.81 (m, 2H), 1.45-1.36 (m, 2H), 1.27-1.12 (m, 4H). ^{13}C NMR (75 MHz, CD_2Cl_2): δ 154.6, 141.7, 140.8, 138.9, 133.2, 131.8, 131.5, 129.7, 128.7, 128.1, 127.9, 126.5, 126.0, 125.2, 124.1, 80.8, 64.6, 55.2, 26.6, 25.1, 24.9, 24.8, 24.7. HRMS (ESI, m/z): Calculated for $C_{27}H_{27}NO_3S$ $(M+H)^+$ 446.1712, found 446.1703.

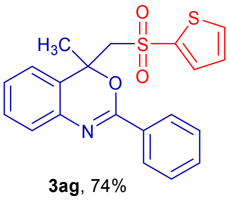

3ag, 74%

4-methyl-4-((phenylsulfonyl)methyl)-2-(thiophen-2-yl)-4*H*-benzo[d][1,3]oxazine (3ag): By following the typical procedure, the product (**3ag**) was isolated as a pale yellow oil, 285 mg (74% yield), from a column chromatography (petroleum ether/ethyl acetate = 9/1). IR (KBr, cm^{-1}): 3062, 2927, 2840, 1625, 1579, 1489, 1317, 1261, 1072. ^{1}H NMR (300 MHz, CD_2Cl_2): δ 7.72 (d, *J* = 7.4 Hz, 2H), 7.64-7.63 (m, 1H), 7.47 (d, *J* = 7.4 Hz, 2H), 7.39-7.35 (m, 2H), 7.29-7.26 (m, 1H), 7.22-7.20 (m, 1H), 7.15-7.11 (m, 2H), 7.06 (d, *J* = 7.8 Hz, 1H), 3.81 (d, *J* = 14.8 Hz, 1H), 3.62 (d, *J* = 14.8 Hz, 1H), 2.08 (s, 3H). ^{13}C NMR (75 MHz, CD_2Cl_2): δ 152.4, 140.7, 138.3, 136.1, 133.5, 130.9, 130.7, 129.6, 129.3, 127.7, 126.9, 126.7, 125.3, 123.2, 78.3, 63.8, 26.9. HRMS (ESI, m/z): Calculated for $C_{20}H_{17}NO_3S_2$ $(M+H)^+$ 384.0650, found 384.0662.

References

1. B. S. Furniss, A. J. Hannaford, P. W. G. Smith and A. R. Tatchell, in Vogel's Textbook of Practical Organic Chemistry, Pearson Education, 5th edn, 1989, ISBN: 978-81-7758-957-3.
2. J. Wu, Y. Zong, C. Zhao, Q. Yan, L. Sun, Y. Li, J. Zhao, Y. Ge and Z. Li, *Org. Biomol. Chem.*, 2019, **17**, 794-797.
3. T. Liu, D. Zheng, Z. Li and J. Wu, *Adv. Synth. Catal.*, 2017, **360**, 865-869.
4. T.-J. He, W.-Q. Zhong and J.-M. Huang, *Chem. Commun.*, 2020, **56**, 2735-2738.

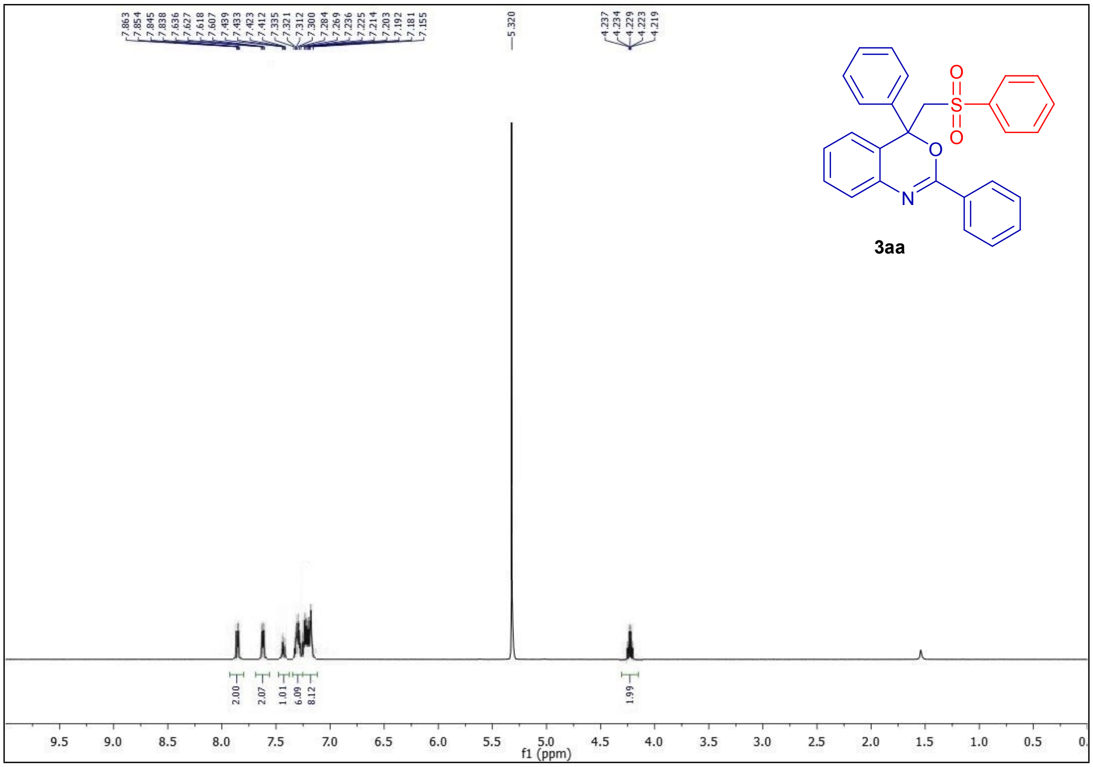

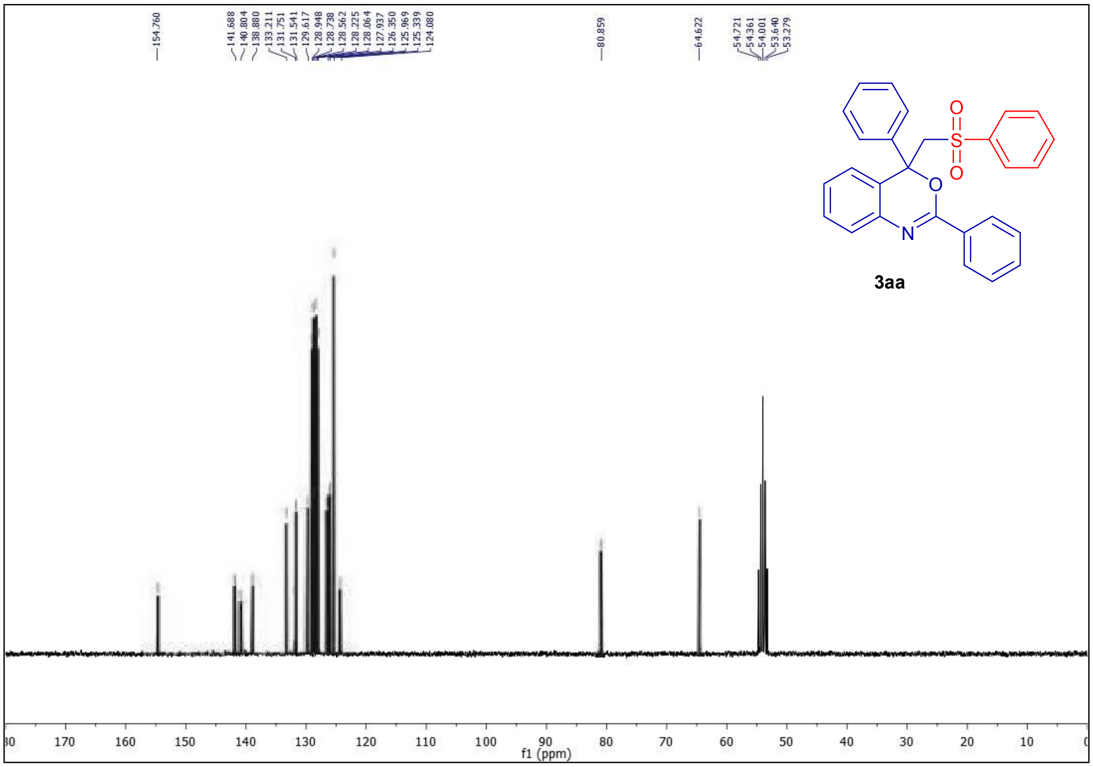

Figure S1. ^{1}H (top, 300 MHz) and ^{13}C (bottom, 75 MHz) NMR spectra of **3aa** in CD_2Cl_2.

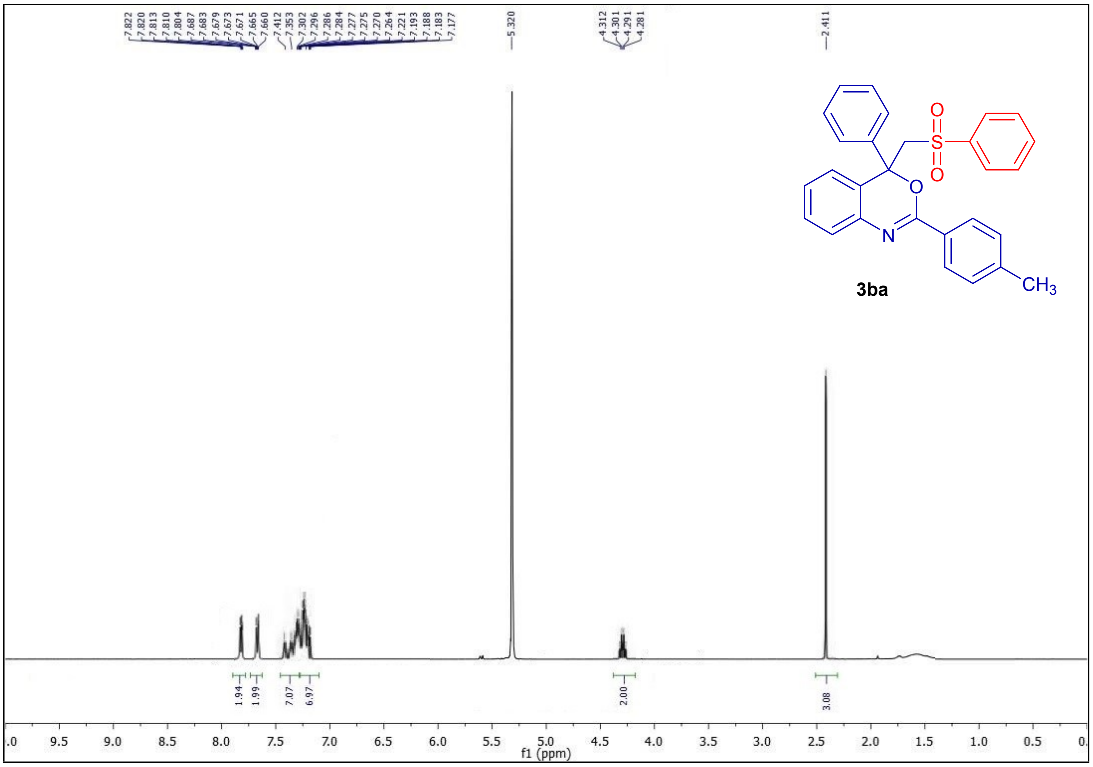

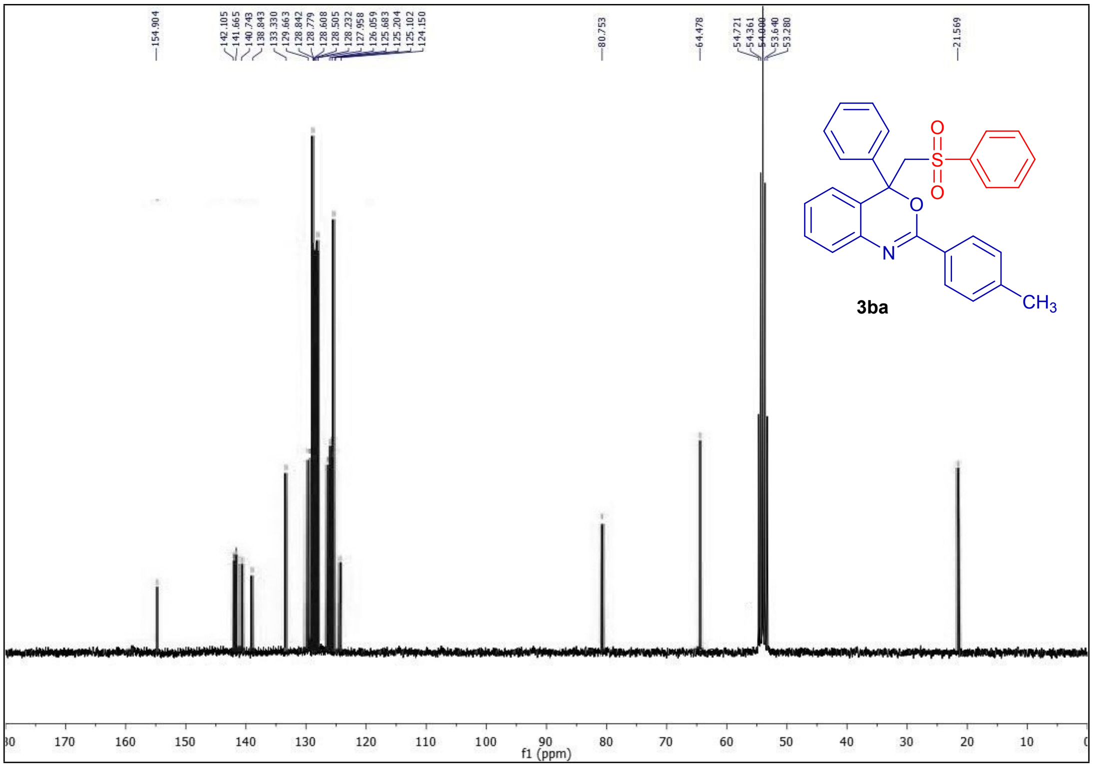

Figure S2. ^{1}H (top, 300 MHz) and ^{13}C (bottom, 75 MHz) NMR spectra of **3ba** in CD_2Cl_2.

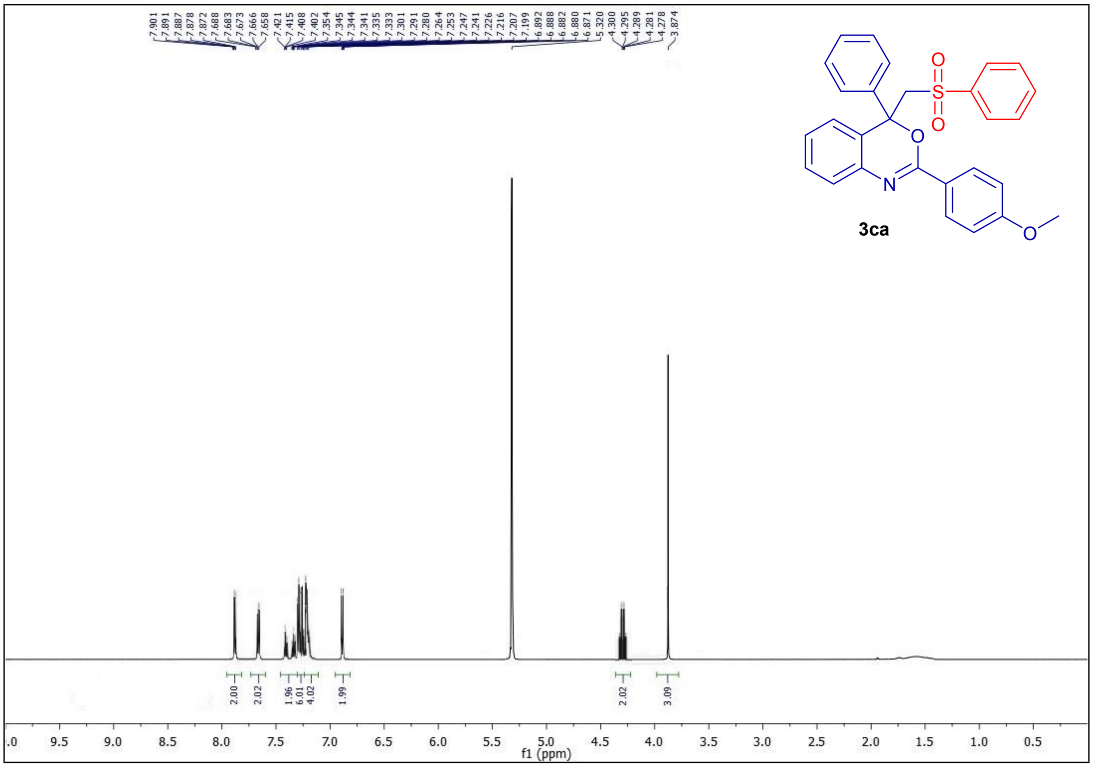

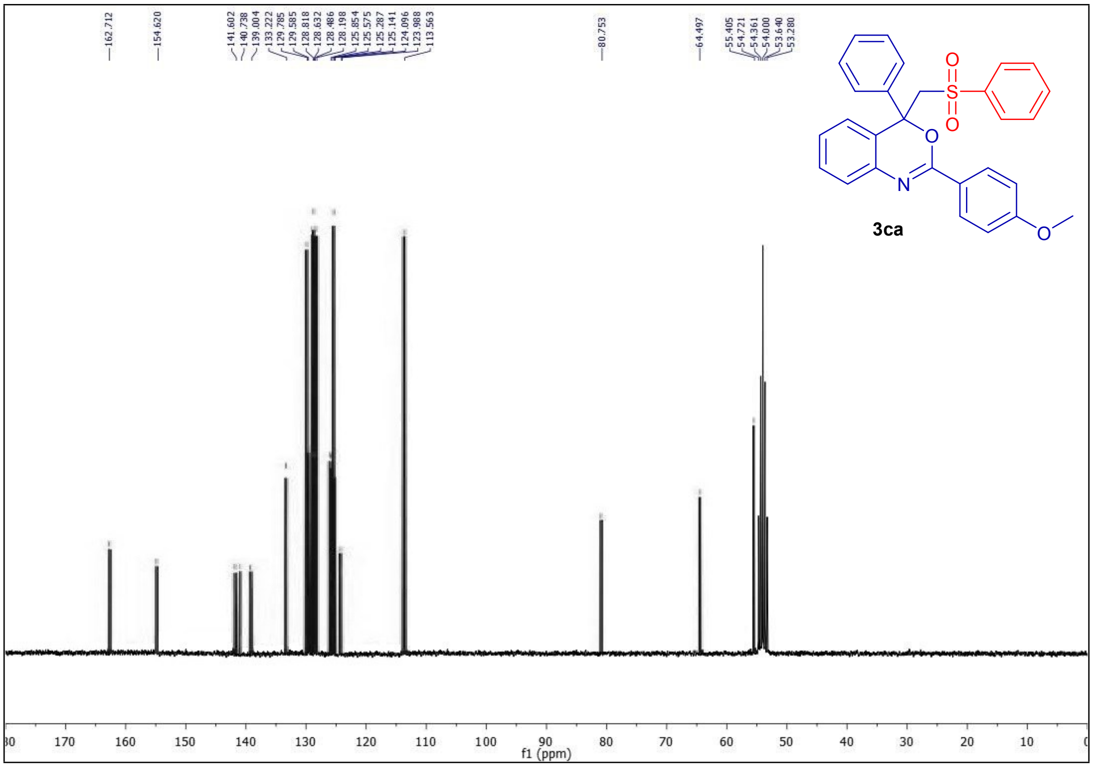

Figure S3. ^{1}H (top, 300 MHz) and ^{13}C (bottom, 75 MHz) NMR spectra of **3ca** in CD_2Cl_2.

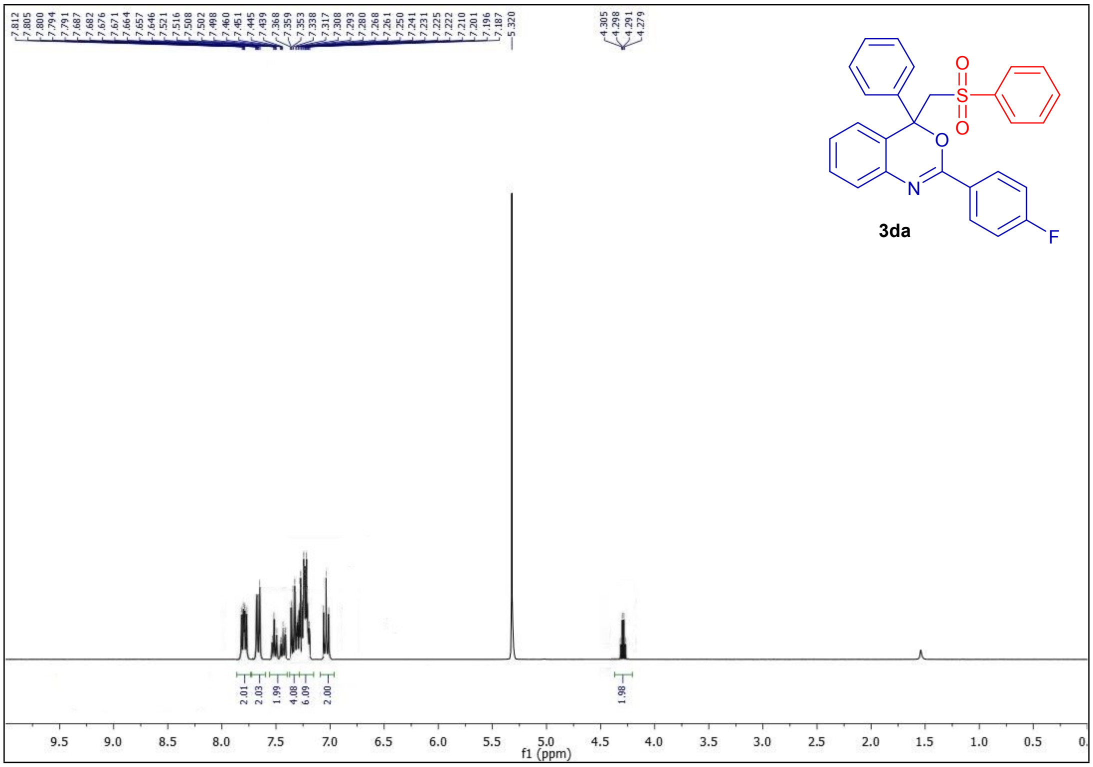

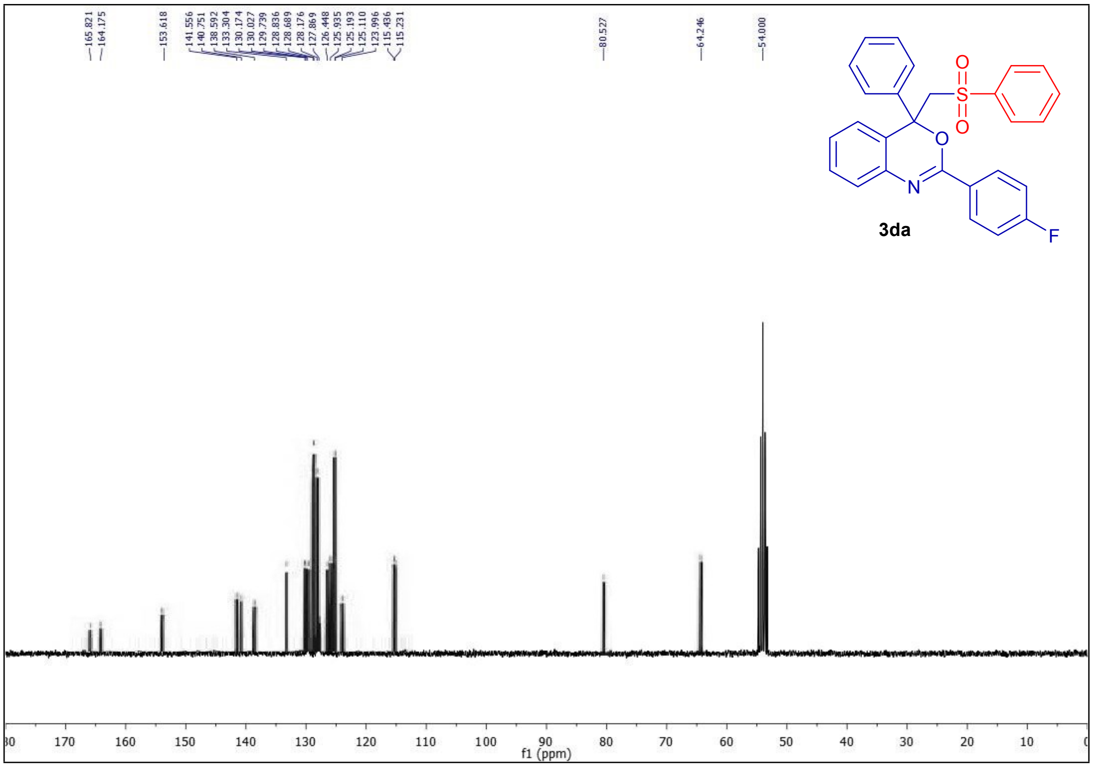

Figure S4. ^{1}H (top, 300 MHz) and ^{13}C (bottom, 75 MHz) NMR spectra of **3da** in CD_2Cl_2.

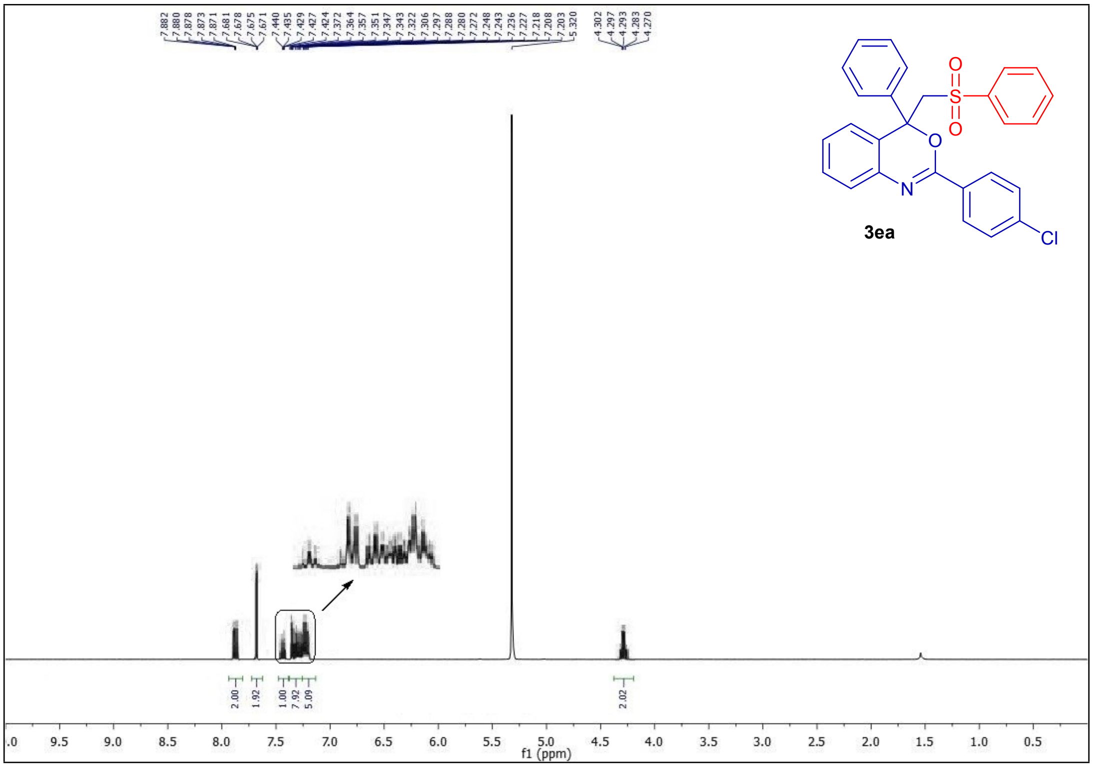

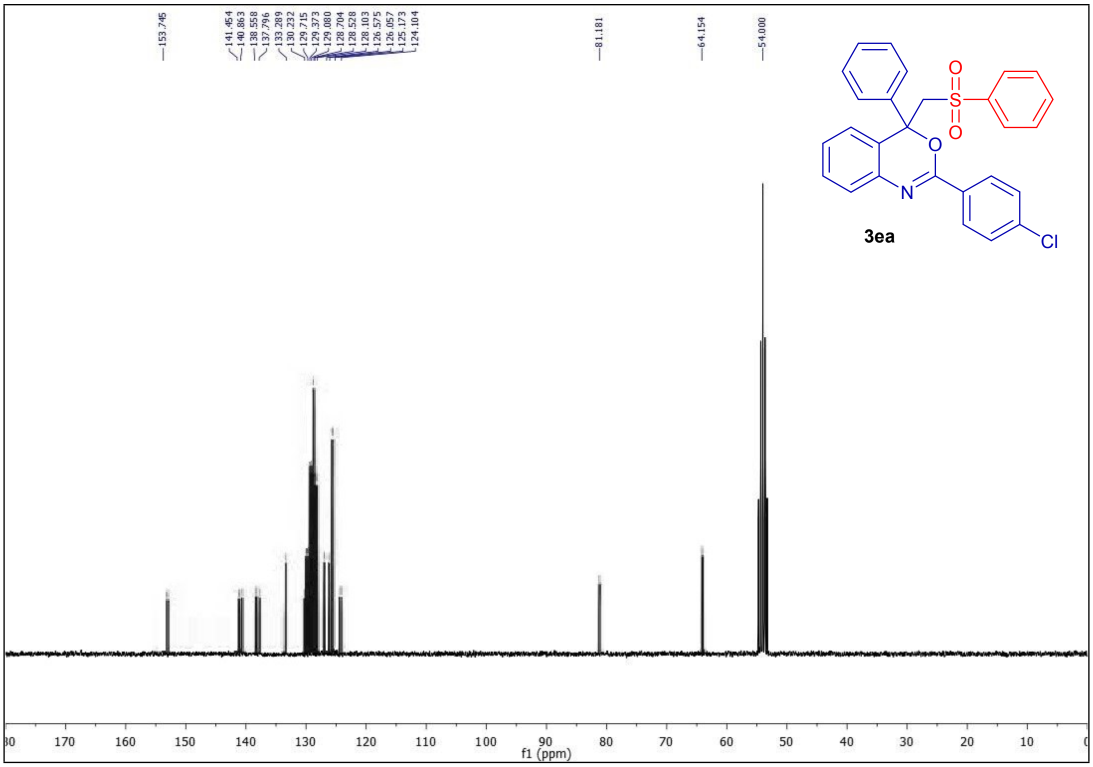

Figure S5. ^{1}H (top, 300 MHz) and ^{13}C (bottom, 75 MHz) NMR spectra of **3ea** in CD_2Cl_2.

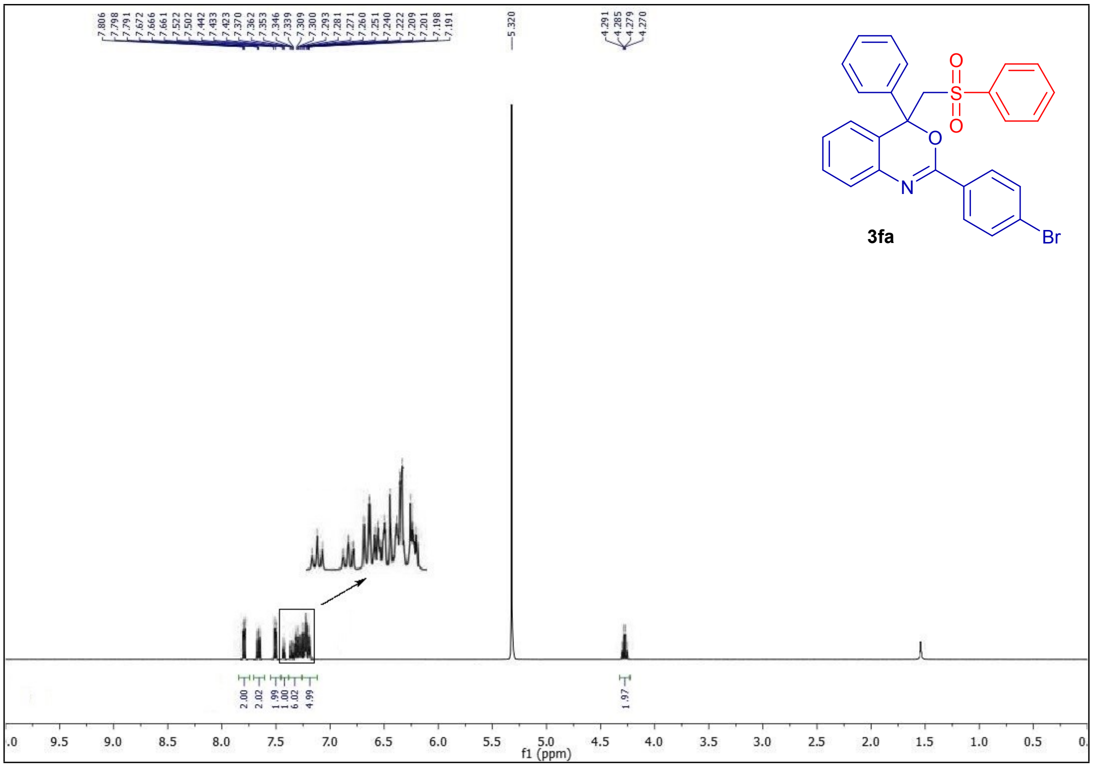

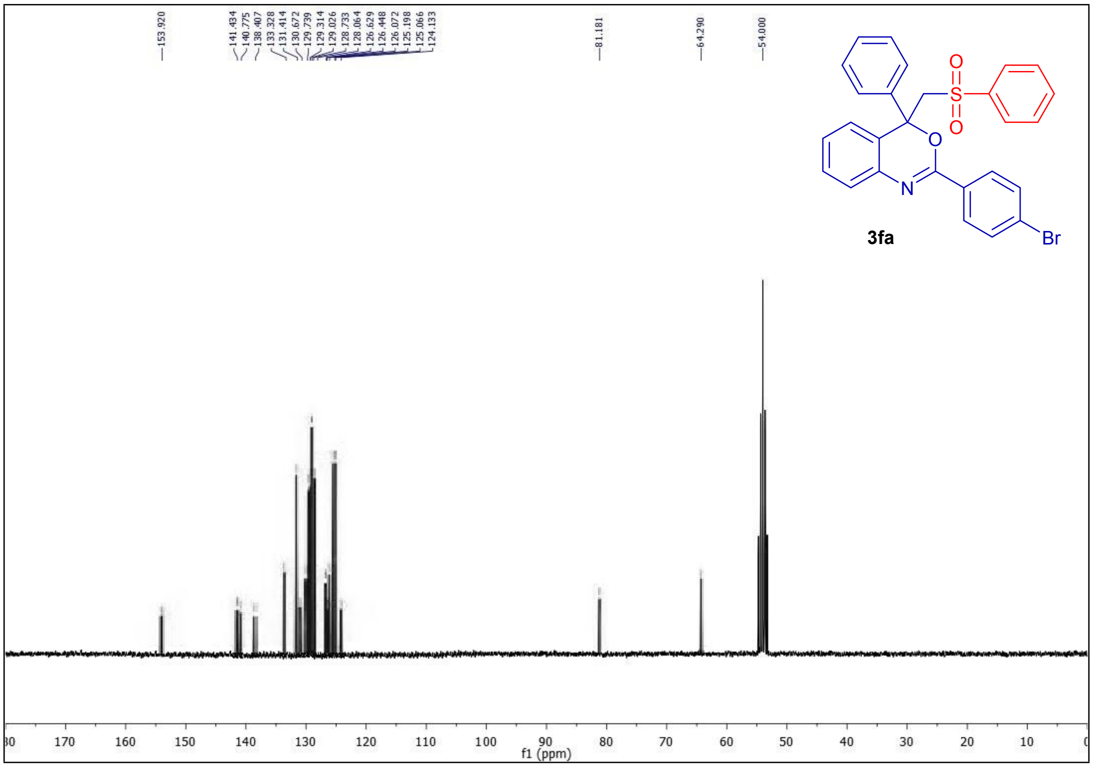

Figure S6. ^{1}H (top, 300 MHz) and ^{13}C (bottom, 75 MHz) NMR spectra of **3fa** in CD_2Cl_2.

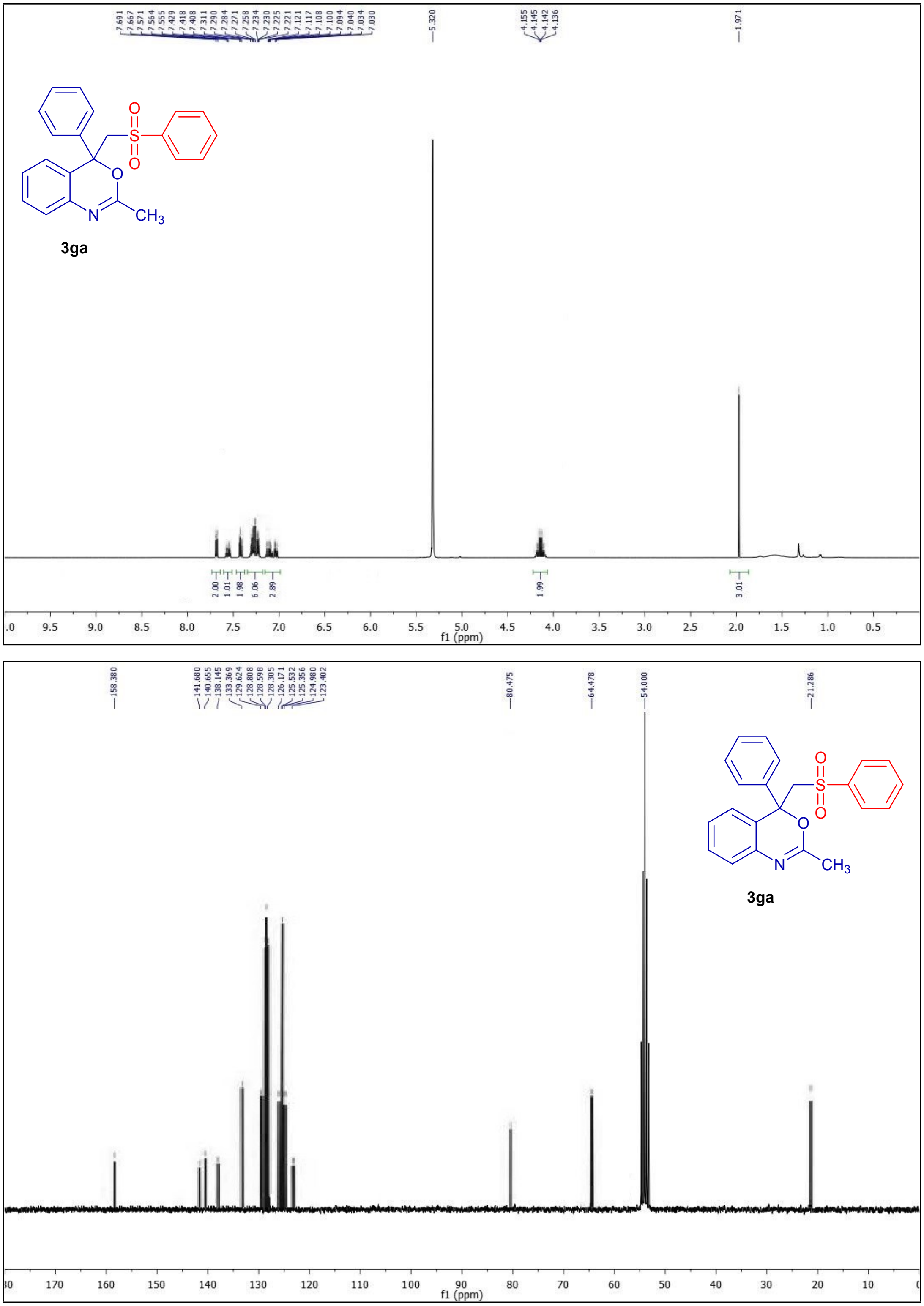

Figure S7. ^{1}H (top, 300 MHz) and ^{13}C (bottom, 75 MHz) NMR spectra of **3ga** in CD_2Cl_2.

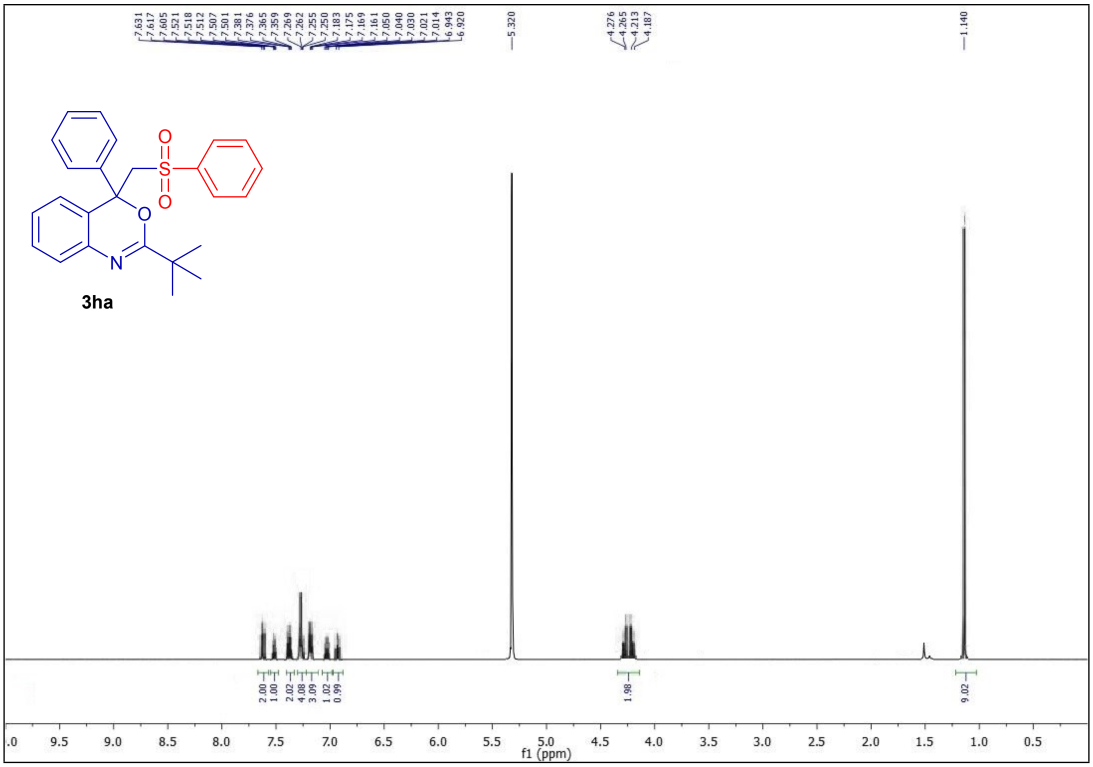

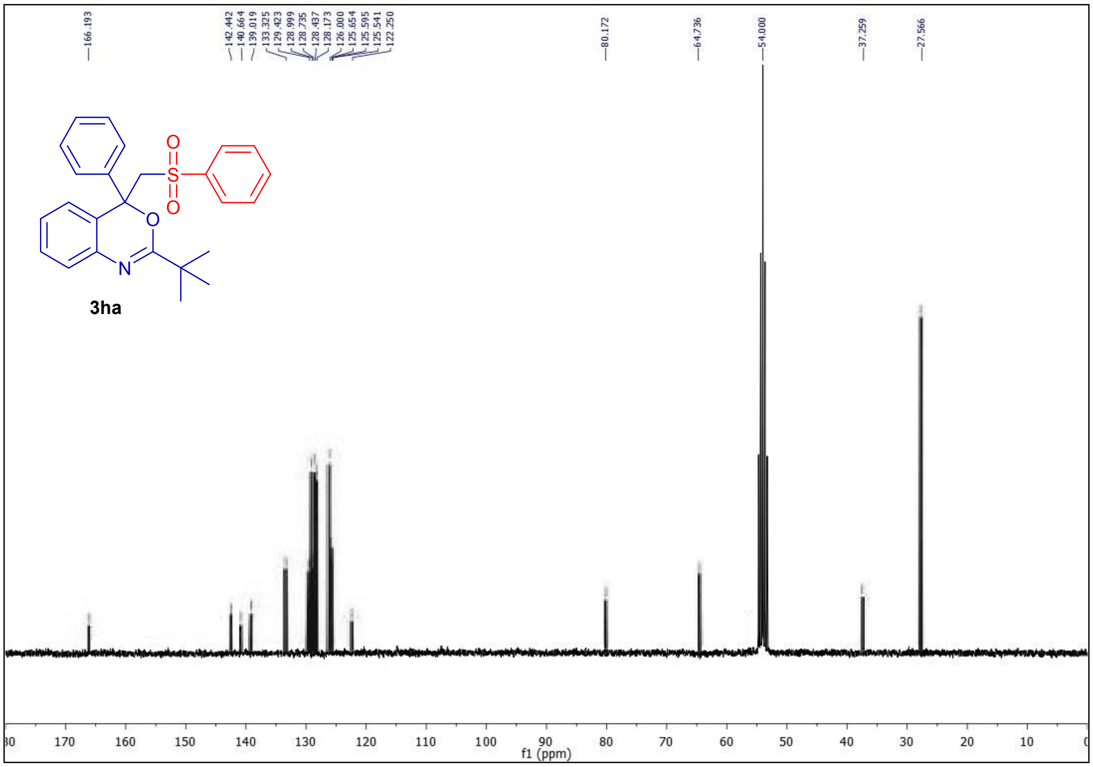

Figure S8. ^{1}H (top, 300 MHz) and ^{13}C (bottom, 75 MHz) NMR spectra of **3ha** in CD_2Cl_2.

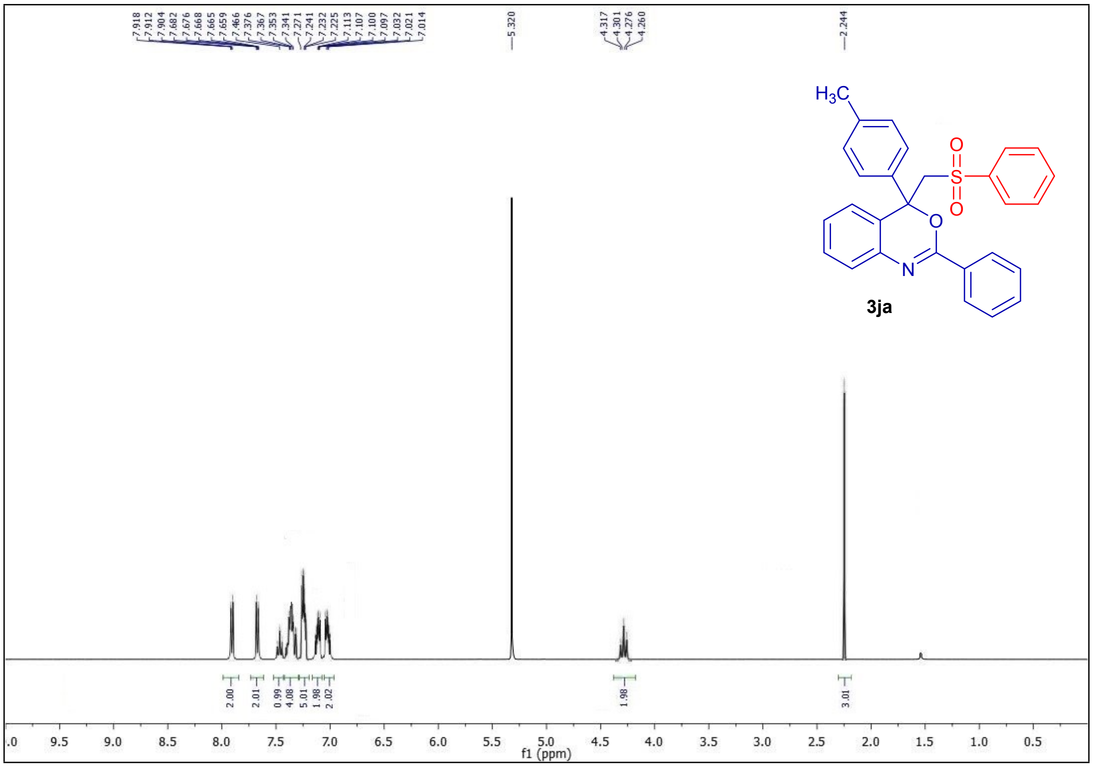

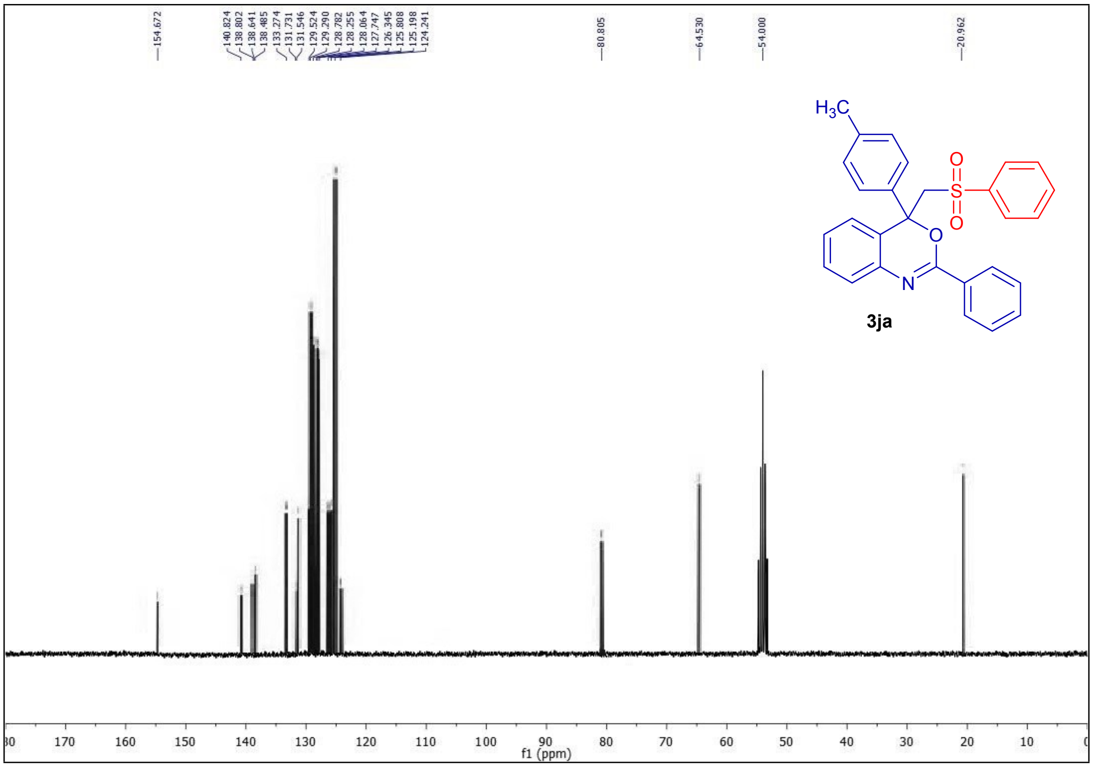

Figure S9. ^{1}H (top, 300 MHz) and ^{13}C (bottom, 75 MHz) NMR spectra of **3ja** in CD_2Cl_2.

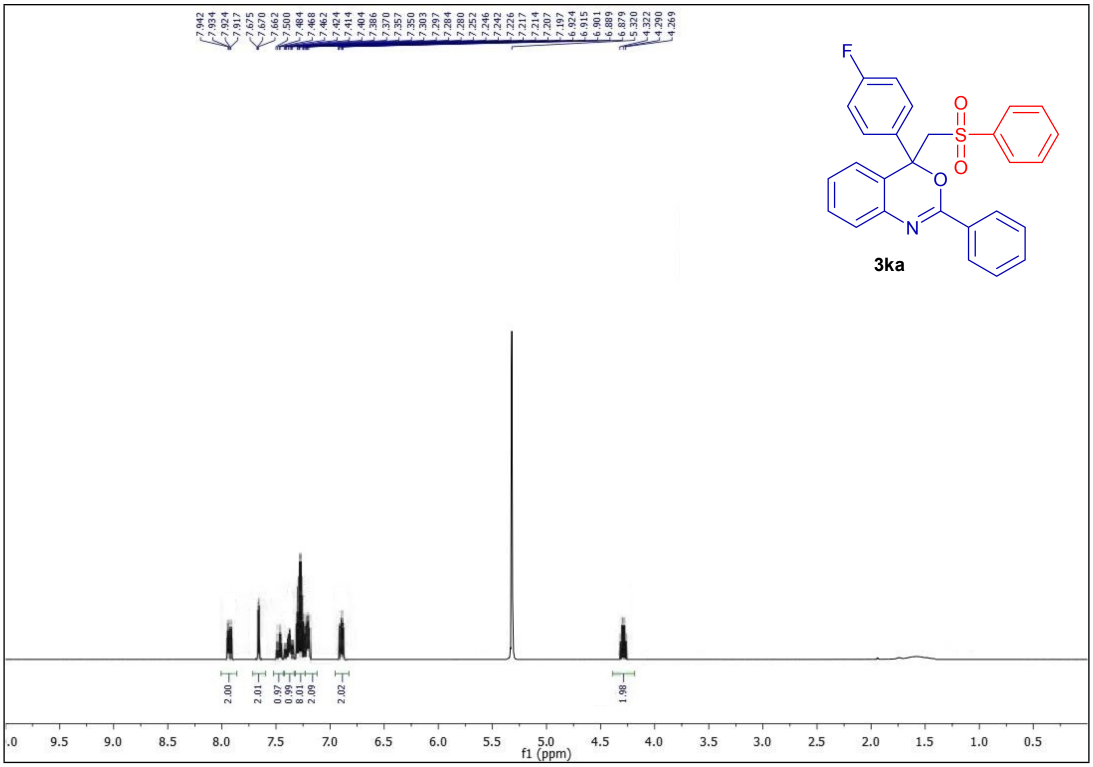

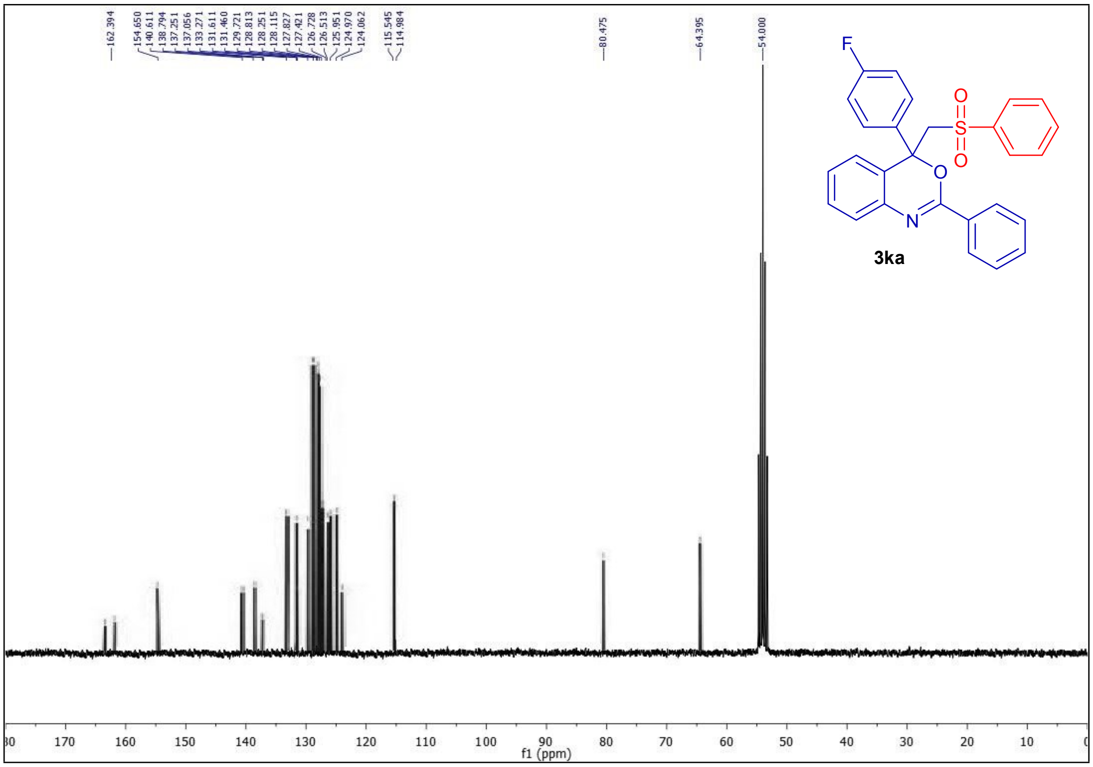

Figure S10. ^{1}H (top, 300 MHz) and ^{13}C (bottom, 75 MHz) NMR spectra of **3ka** in CD_2Cl_2.

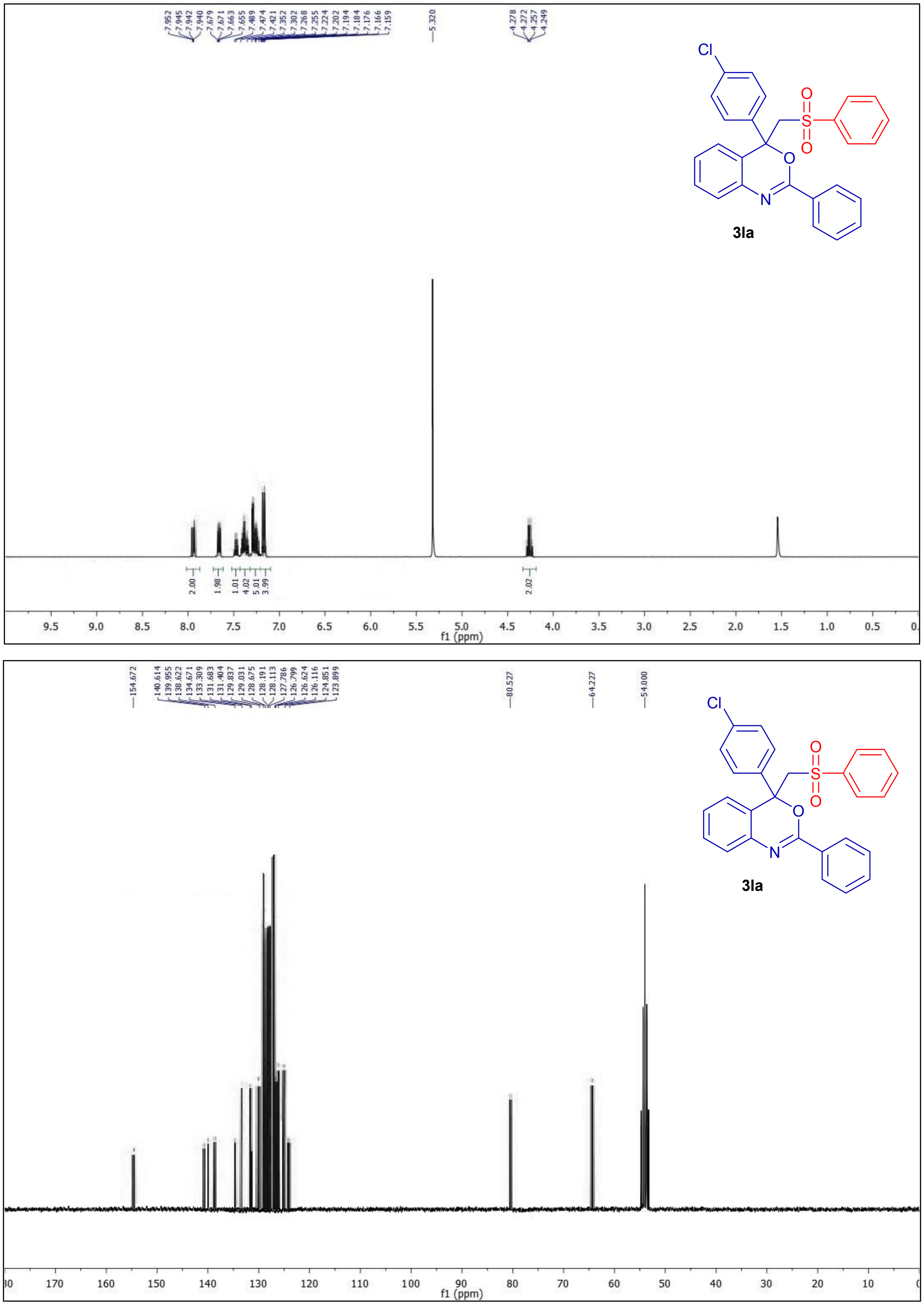

Figure S11. ^{1}H (top, 300 MHz) and ^{13}C (bottom, 75 MHz) NMR spectra of **3la** in CD_2Cl_2.

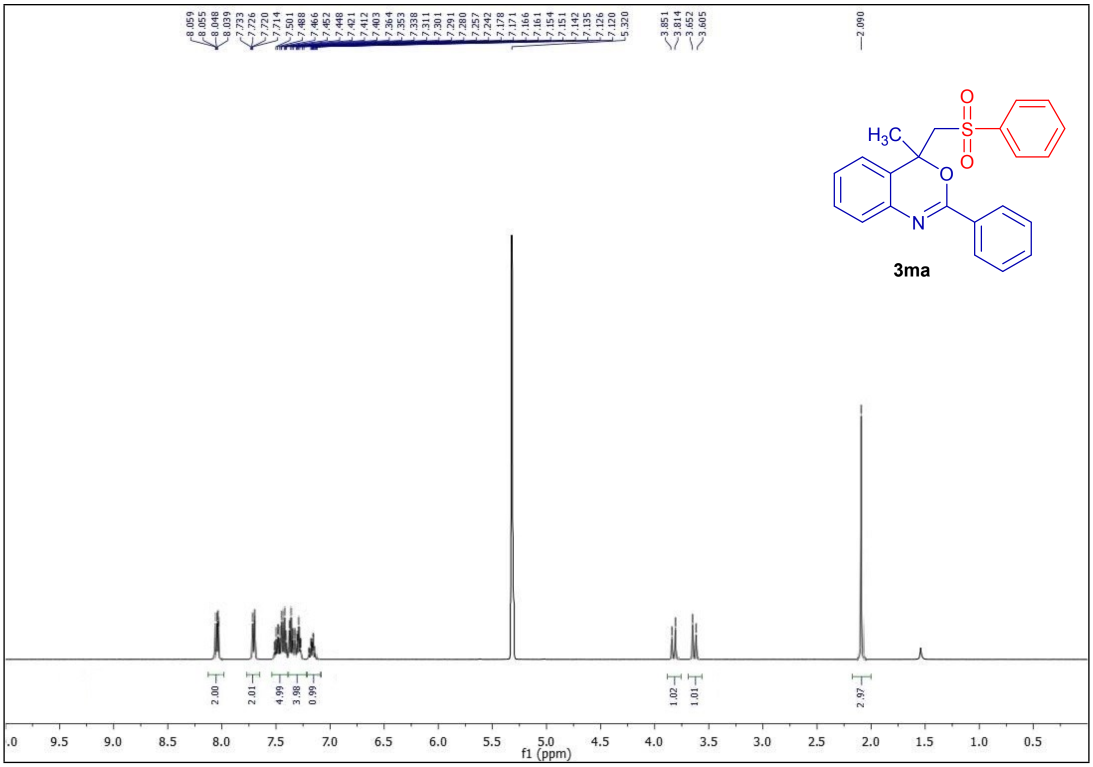

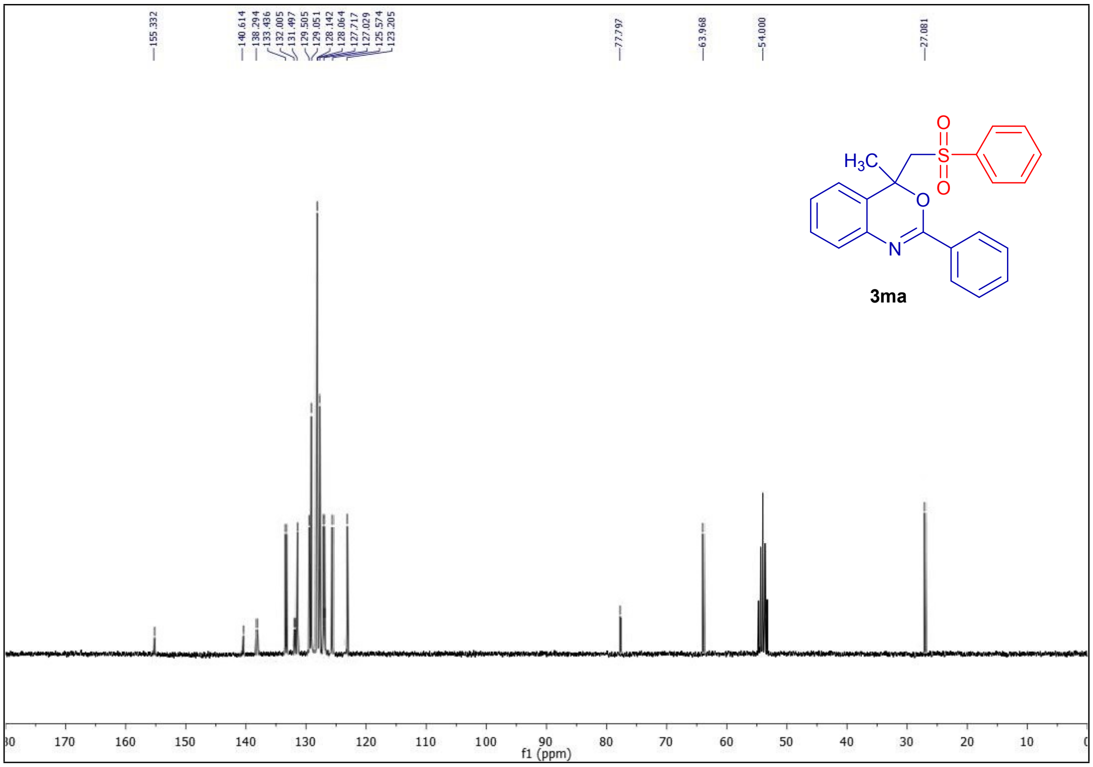

Figure S12. ^{1}H (top, 300 MHz) and ^{13}C (bottom, 75 MHz) NMR spectra of **3ma** in CD_2Cl_2.

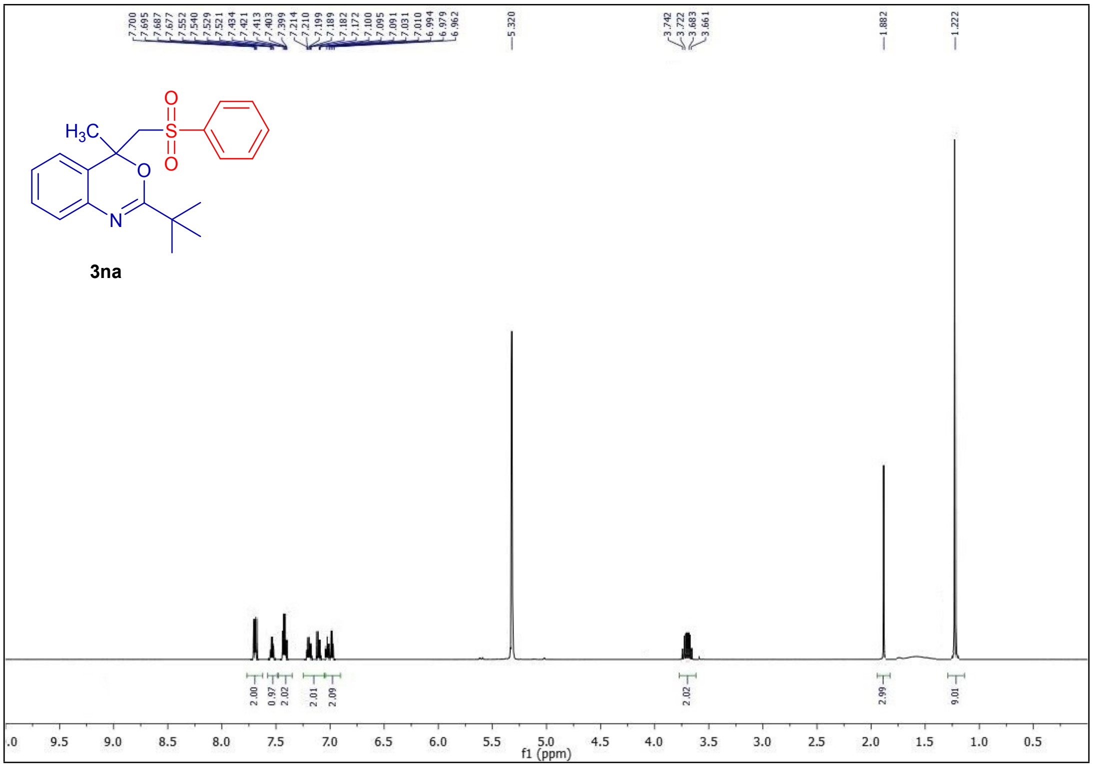

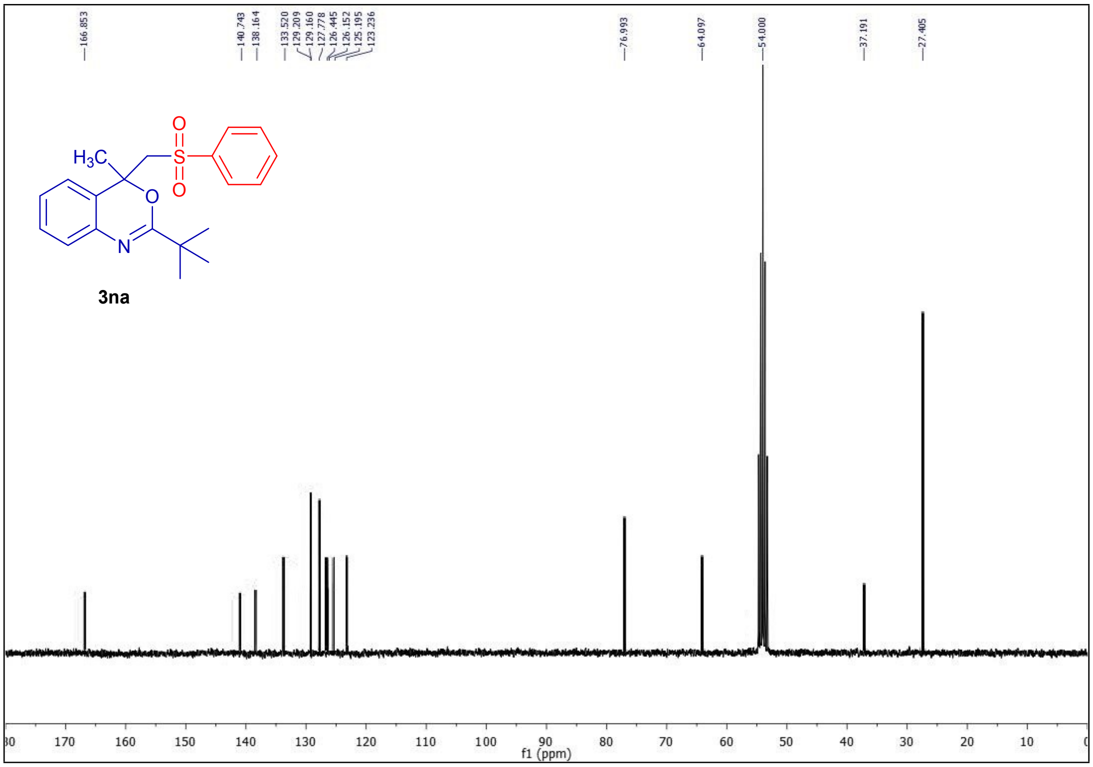

Figure S13. ^{1}H (top, 300 MHz) and ^{13}C (bottom, 75 MHz) NMR spectra of **3na** in CD_2Cl_2.

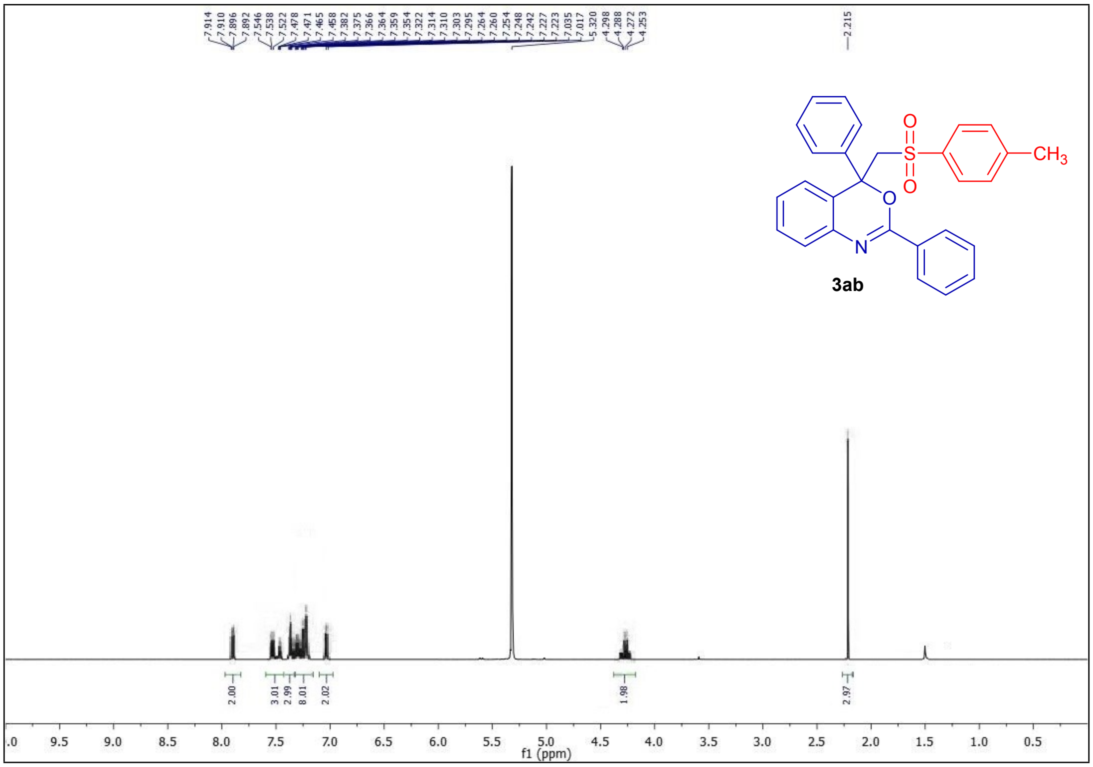

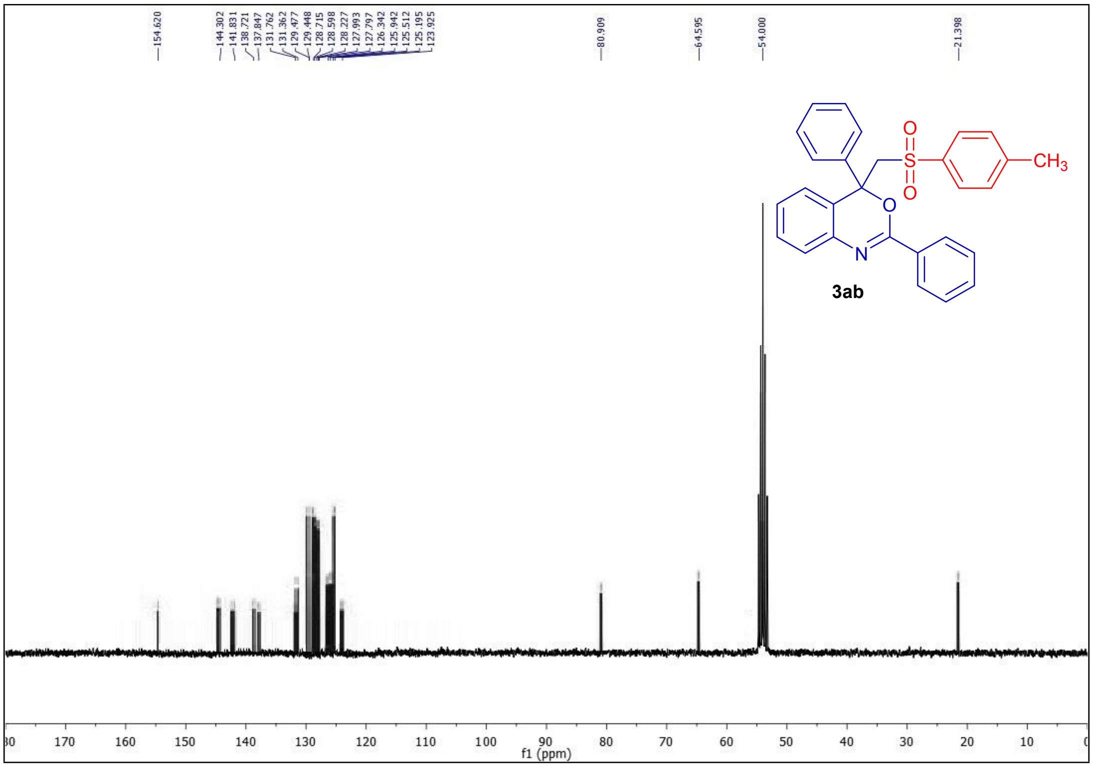

Figure S14. ^{1}H (top, 300 MHz) and ^{13}C (bottom, 75 MHz) NMR spectra of **3ab** in CD_2Cl_2.

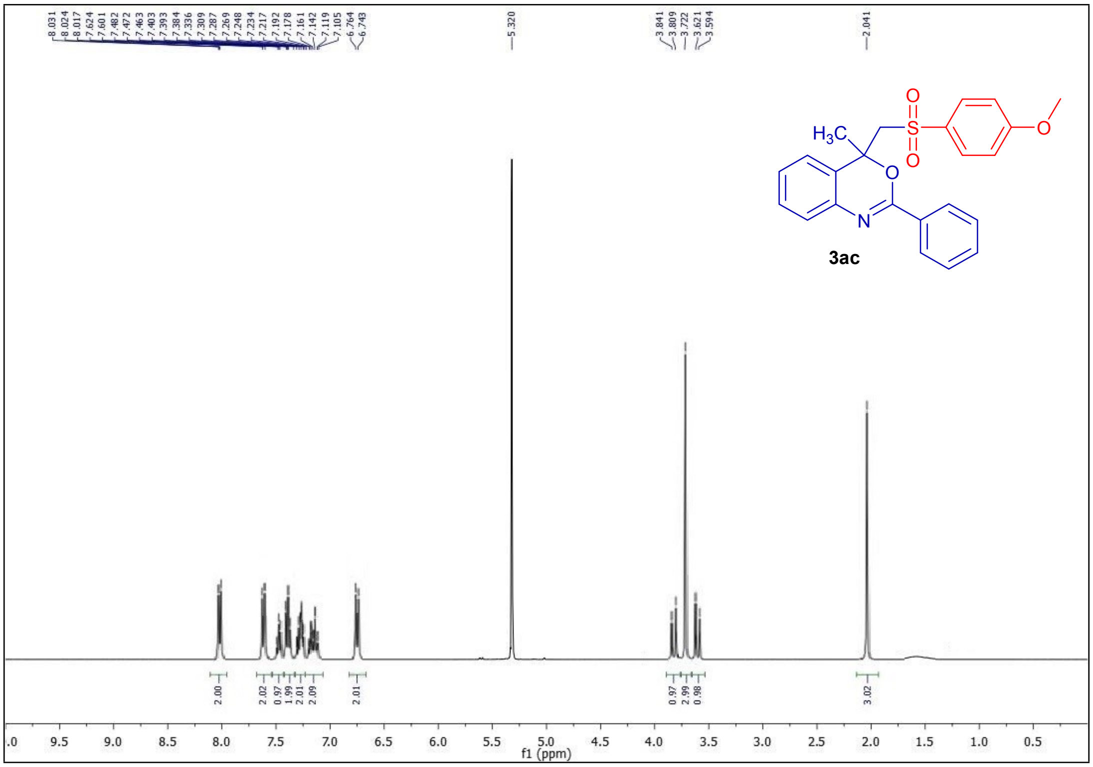

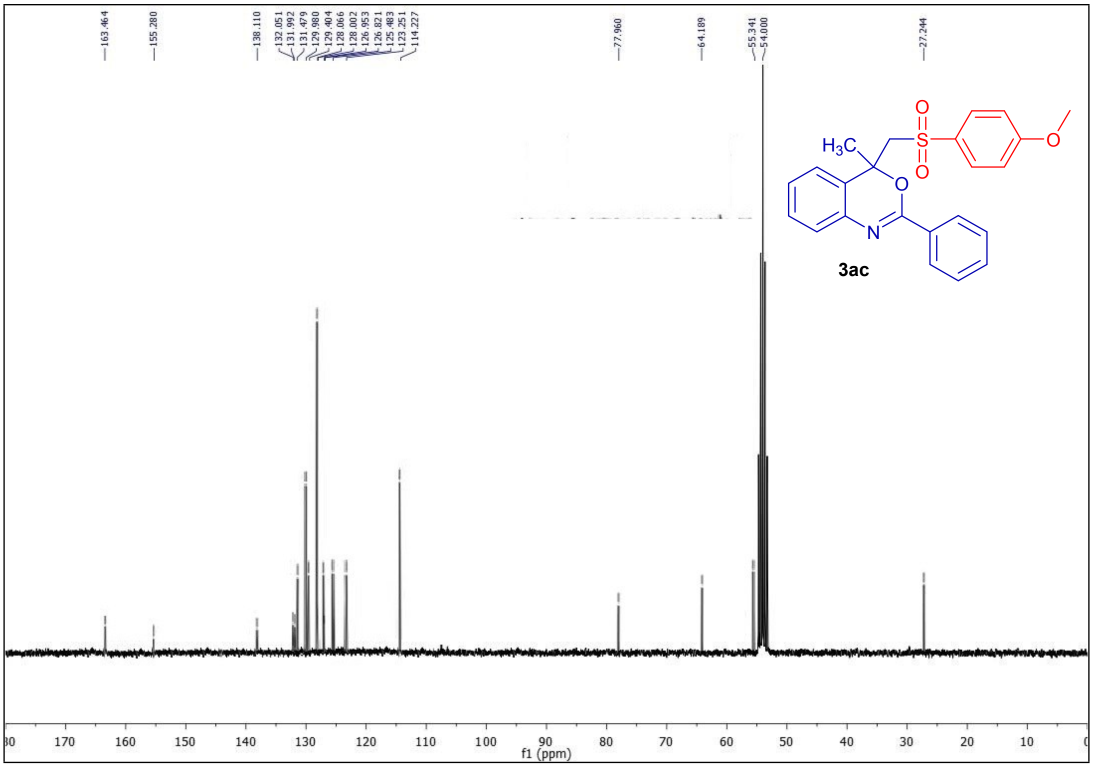

Figure S15. ^{1}H (top, 300 MHz) and ^{13}C (bottom, 75 MHz) NMR spectra of **3ac** in CD_2Cl_2.

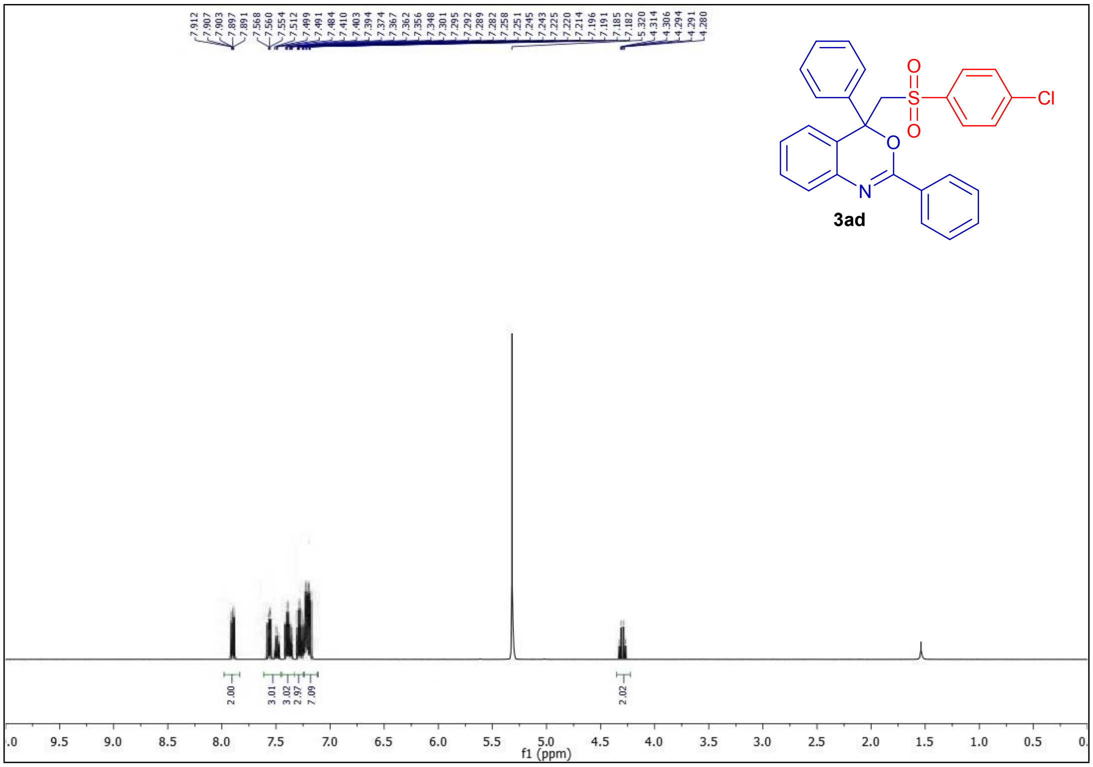

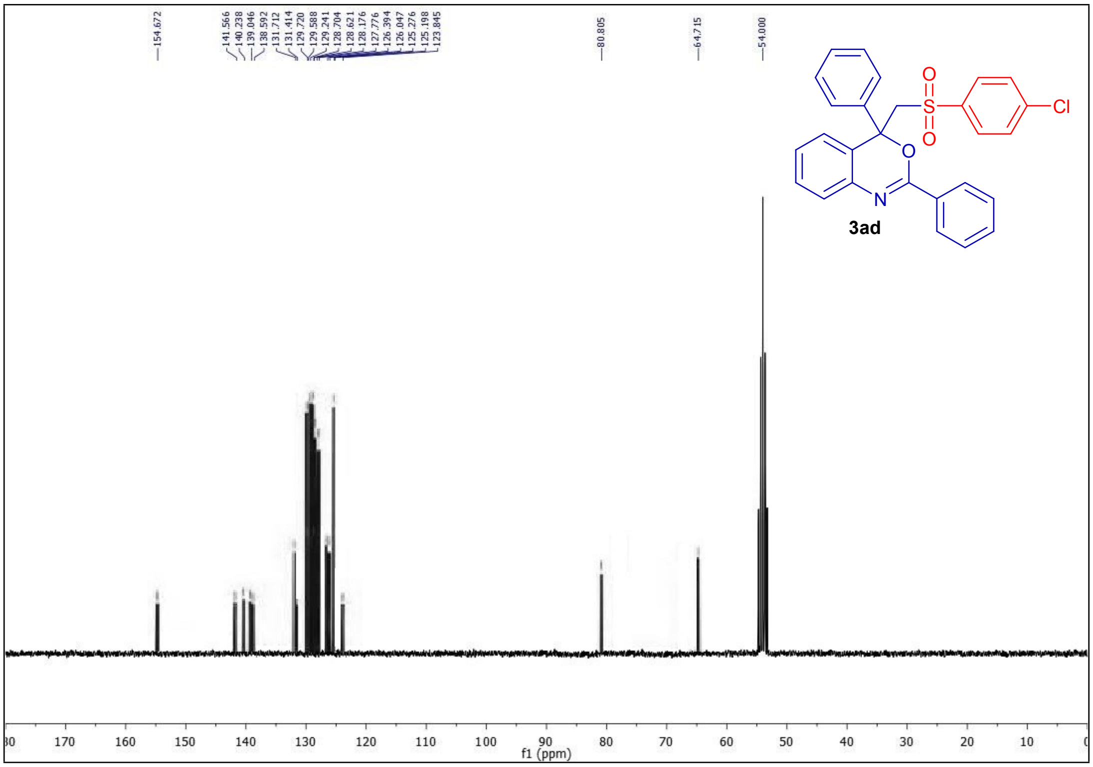

Figure S16. ^{1}H (top, 300 MHz) and ^{13}C (bottom, 75 MHz) NMR spectra of **3ad** in CD_2Cl_2.

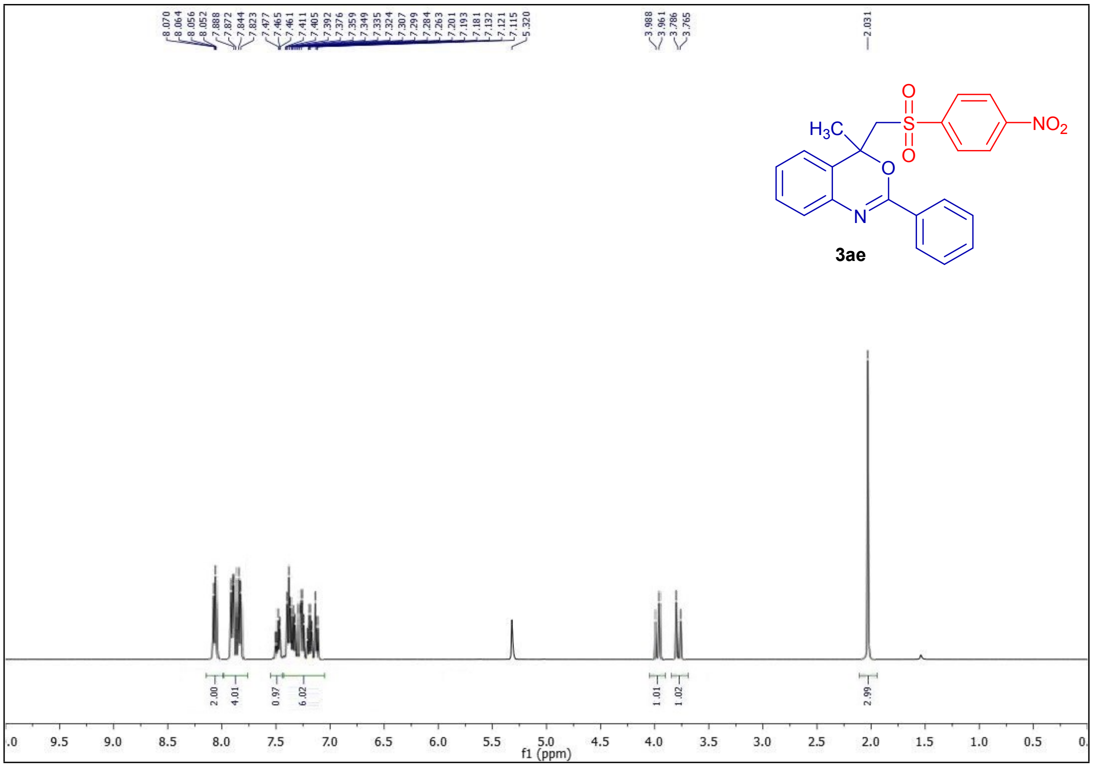

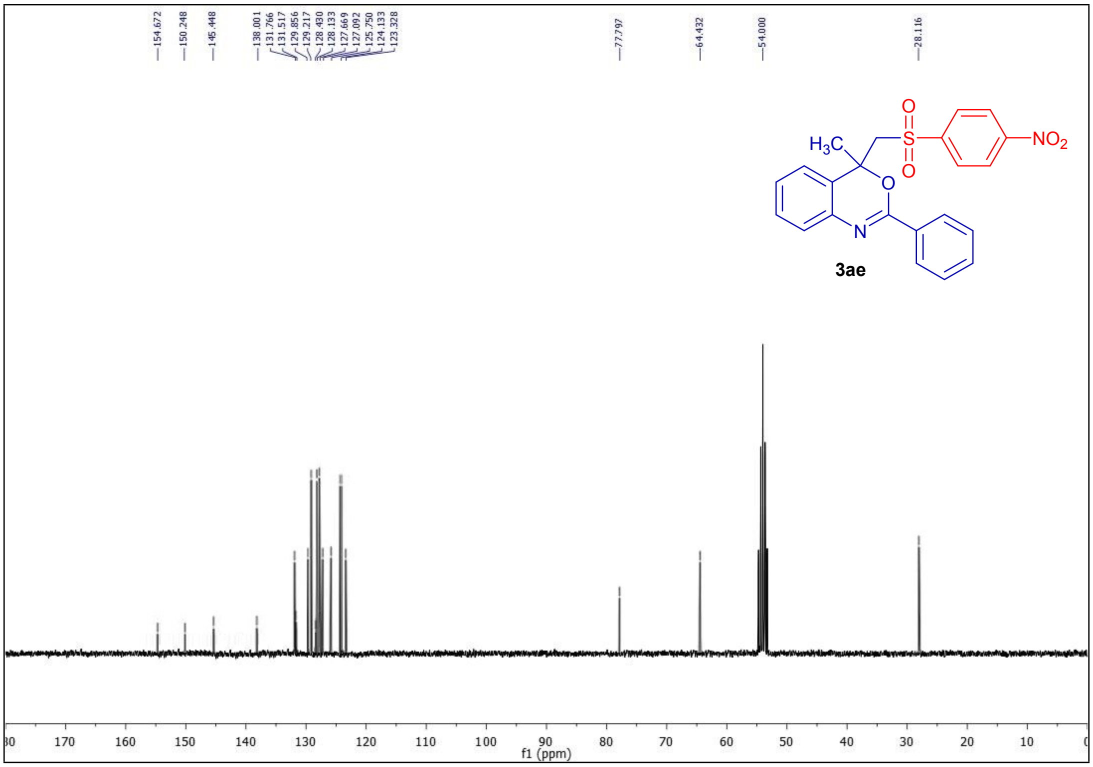

Figure S17. ^{1}H (top, 300 MHz) and ^{13}C (bottom, 75 MHz) NMR spectra of **3ae** in CD_2Cl_2.

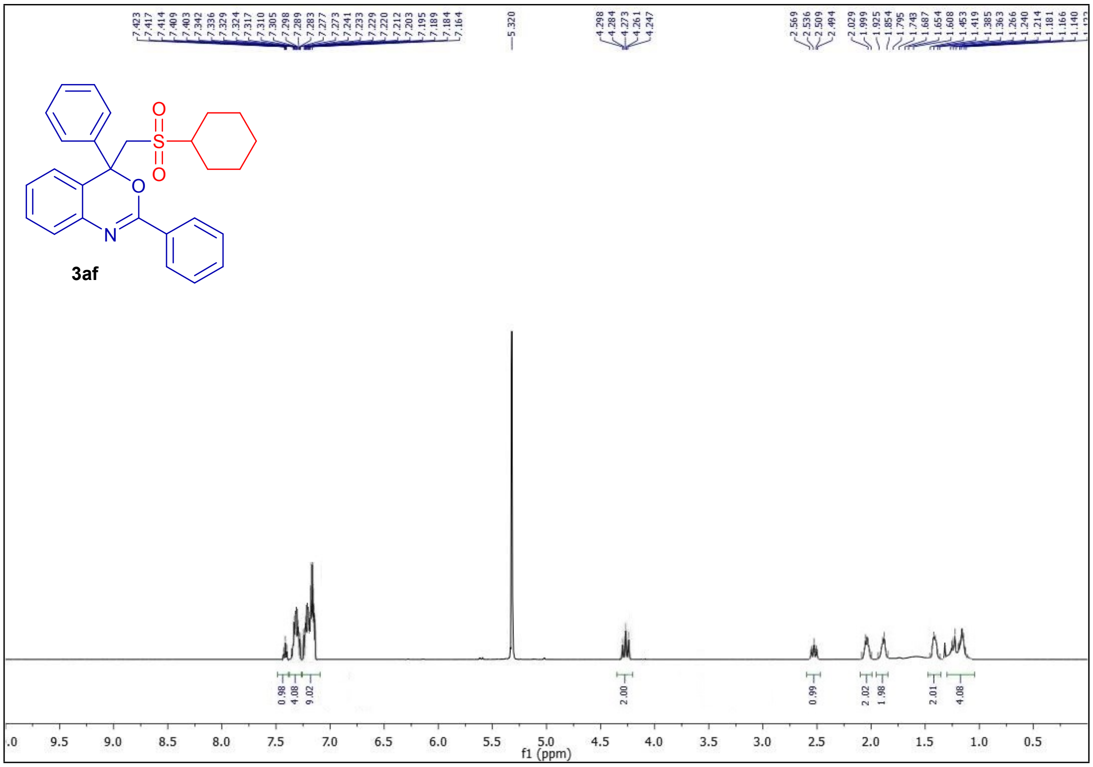

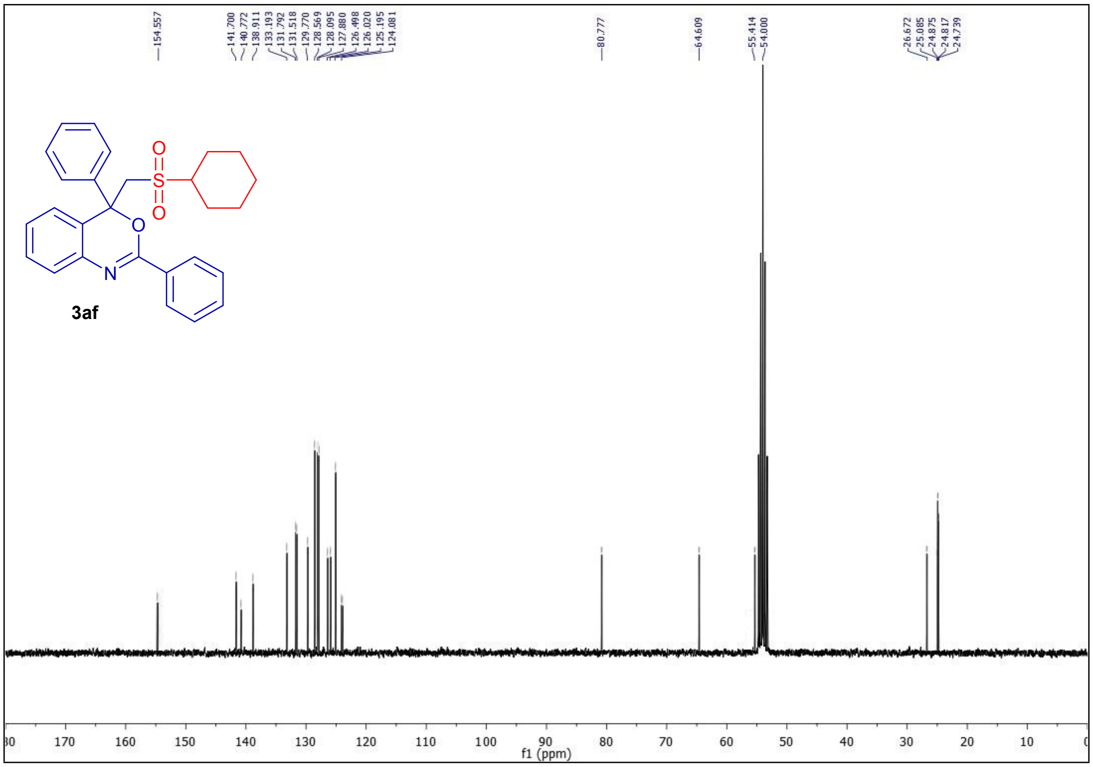

Figure S18. ^{1}H (top, 300 MHz) and ^{13}C (bottom, 75 MHz) NMR spectra of **3af** in CD_2Cl_2.

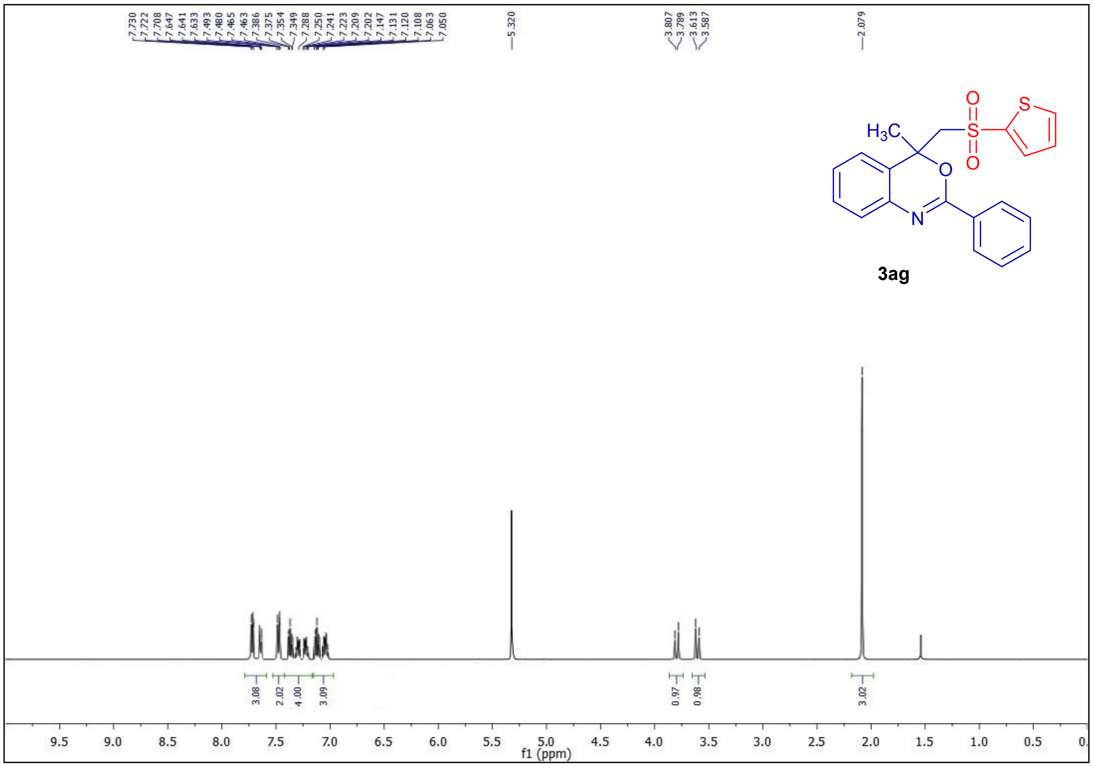

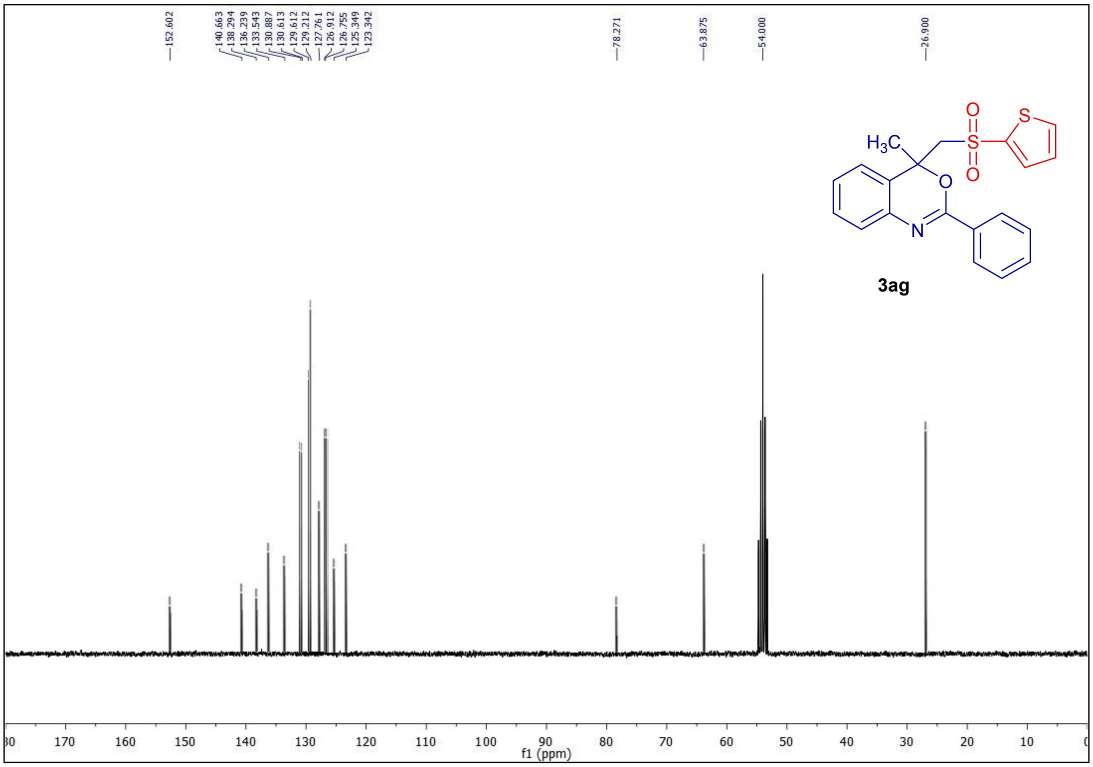

Figure S19. ^{1}H (top, 300 MHz) and ^{13}C (bottom, 75 MHz) NMR spectra of **3ag** in CD_2Cl_2.
